# Grand Challenges in global eye health: a global prioritisation process using Delphi method

**DOI:** 10.1016/S2666-7568(21)00302-0

**Published:** 2022-01

**Authors:** Jacqueline Ramke, Jennifer R Evans, Esmael Habtamu, Nyawira Mwangi, Juan Carlos Silva, Bonnielin K Swenor, Nathan Congdon, Hannah B Faal, Allen Foster, David S Friedman, Stephen Gichuhi, Jost B Jonas, Peng T Khaw, Fatima Kyari, Gudlavalleti V S Murthy, Ningli Wang, Tien Y Wong, Richard Wormald, Mayinuer Yusufu, Hugh Taylor, Serge Resnikoff, Sheila K West, Matthew J Burton, Grand Challenges in Global Eye Health study group, Ada Aghaji, Adeyemi T Adewole, Adrienne Csutak, Ahmad Shah Salam, Ala Paduca, Alain M Bron, Alastair K Denniston, Alberto Lazo Legua, Aldiana Halim, Alemayehu Woldeyes Tefera, Alice Mwangi, Alicia J Jenkins, Amanda Davis, Amel Meddeb-Ouertani, Amina H Wali, Ana G Palis, Ana Bastos de Carvalho, Anagha Joshi, Andreas J Kreis, Andreas Mueller, Andrew Bastawrous, Andrew Cooper, Andrew F Smith, Andrzej Grzybowski, Anitha Arvind, Anne M Karanu, Anne O Orlina, Anthea Burnett, Aryati Yashadhana, Asela P Abeydeera, Aselia Abdurakhmanova, Ashik Mohamed, Ashish Bacchav, Ashlie Bernhisel, Aubrey Walton Webson, Augusto Azuara-Blanco, Ava Hossain, Bayazit Ilhan, Bella Assumpta Lucienne, Benoit Tousignant, Bindiganavale R Shamanna, Boateng Wiafe, Brigitte Mueller, Cagatay Caglar, Caleb Mpyet, Carl H Abraham, Carol Y Cheung, Cassandra L Thiel, Catherine L Jan, Chike Emedike, Chimgee Chuluunkhuu, Chinomso Chinyere, Christin Henein, Clare E Gilbert, Covadonga Bascaran, Cristina Elena Nitulescu, Daksha Patel, Damodar Bachani, Daniel Kiage, Daniel Etya'ale, David Dahdal, Dawn Woo Lawson, Denise Godin, Dennis G Nkanga, Dennis M Ondeyo, Donna O'Brien, Dorothy M Mutie, Ebtisam S K Alalawi, Eduardo Mayorga, Effendy Bin Hashim, Elham Ashrafi, Elizabeth Andrew Kishiki, Elizabeth Kurian, Fabrizio D'Esposito, Faith Masila, Fernando Yaacov Pena, Fortunat Büsch, Fotis Topouzis, Francesco Bandello, Funmilayo J Oyediji, Gabriele Thumann, Gamal Ezz Elarab, Gatera Fiston Kitema, Gerhard Schlenther, Gertrude Oforiwa Fefoame, Gillian M Cochrane, Guna Laganovska, Haroon R Awan, Harris M Ansari, Heiko Philippin, Helen Burn, Helen Dimaras, Helena P Filipe, Henrietta I Monye, Himal Kandel, Hoby Lalaina Randrianarisoa, Iain Jones, Ian E Murdoch, Ido Didi Fabian, Imran A Khan, Indra P Sharma, Islam Elbeih, Islay Mactaggart, J Carlos Pastor, Jan E E Keunen, Jane A Ohuma, Jason Pithuwa Nirwoth, Jaouad Hammou, Jayme R Vianna, Jean-eudes Biao, Jennifer M Burr, Jeremy D Keenan, Jess Blijkers, Joanna M Black, Joao Barbosa Breda, Joao M Furtado, John C Buchan, John G Lawrenson, John H Kempen, Joshua R Ehrlich, Judith Stern, Justine H Zhang, Kadircan H Keskinbora, Karin M Knoll, Karl Blanchet, Katrina L Schmid, Koichi Ono, Kolawole Ogundimu, Komi Balo, Kussome Paulin Somda, Kwame Yeboah, Kwesi N Amissah-Arthur, Leone Nasehi, Lene Øverland, Lingam Vijaya, Lisa Keay, Lisa M Hamm, Lizette Mowatt, Lloyd C M Harrison-Williams, Lucia Silva, Luigi Bilotto, Manfred Mörchen, Mansur Rabiu, Marcia Zondervan, Margarida Chagunda, Maria Teresa Sandinha, Mariano Yee Melgar, Marisela Salas Vargas, Mark D Daniell, Marzieh Katibeh, Matt Broom, Megan E Collins, Mehmet Numan Alp, Michael A Kwarteng, Michael Belkin, Michael Gichangi, Michelle Sylvanowicz, Min Wu, Miriam R Cano, Mohammad Shalaby, Mona Duggal, Moncef Khairallah, Muhammed Batur, Mukharram M Bikbov, Muralidhar Ramappa, Nagaraju Pamarathi, Naira Khachatryan, Nasiru Muhammad, Neil Kennedy, Neil Murray, Nicholas A V Beare, Nick Astbury, Nicole A Carnt, Nigel A St Rose, Nigel H Barker, Niranjan K Pehere, Nkechinyere J Uche, Noemi Lois, Oluwaseun O Awe, Oscar J Mujica, Oteri E Okolo, Padmaja Kumari Rani, Paisan Ruamviboonsuk, Papa Amadou Ndiaye, Parami Dhakhwa, Pavel Rozsival, Pearl K Mbulawa, Pearse A Keane, Pete R Jones, Peter Holland, Phanindra Babu Nukella, Philip I Burgess, Pinar Aydin O'Dwyer, Prabhath Piyasena, Pradeep Bastola, Priya Morjaria, Qais Nasimee, Raizza A T Rambacal, Rajdeep Das, Rajiv B Khandekar, Rajvardhan Azad, Ramona Bashshur, Raúl A R C Sousa, Rebecca Oenga, Reeta Gurung, Robert Geneau, Robert J Jacobs, Robert P Finger, Robyn H Guymer, Rodica Sevciuc, Rohit C Khanna, Ronnie George, Ronnie Graham, Ryo Kawasaki, S May Ho, Sailesh Kumar Mishra, Sandeep Buttan, Sandra S Block, Sandra Talero, Sangchul Yoon, Sanil Joseph, Sare Safi, Sarity Dodson, Sergio R Munoz, Seydou Bakayoko, Seyed Farzad Mohammadi, Shabir Ahmad Muez, Shahina Pardhan, Shelley Hopkins, Shwu-Jiuan Sheu, Sidi Mohamed Coulibaly, Silvana A Schellini, Simon Arunga, Simon R Bush, Sobha Sivaprasad, Solange R Salomao, Srinivas Marmamula, Stella N Onwubiko, Stuti L Misra, Subeesh Kuyyadiyil, Sucheta Kulkarni, Sudarshan khanal, Sumrana Yasmin, Suzana Nikolic Pavljasevic, Suzanne S Gilbert, Tasanee Braithwaite, Tatiana Ghidirimschi, Thulasiraj Ravilla, Timothy R Fricke, Tiziana Cogliati, Tsehaynesh Kassa, Tunde Peto, Ute Dibb, Van C Lansingh, Victor H Hu, Victoria M Sheffield, Wanjiku Mathenge, William H Dean, Winifred Nolan, Yoshimune Hiratsuka, Yousaf Jamal Mahsood, Yuddha Sapkota

**Affiliations:** a International Centre for Eye Health, London School of Hygiene & Tropical Medicine, London, UK; b School of Optometry and Vision Science, University of Auckland, Auckland, New Zealand; c Centre for Public Health, Queen's University Belfast, Belfast, UK; d Eyu-Ethiopia: Eye Health Research, Training and Service Centre, Bahirdar, Ethiopia; e Kenya Medical Training College, Nairobi, Kenya; f Pan American Health Organization, Bogotá, Colombia; g Dana Center for Preventive Ophthalmology, Johns Hopkins University, Baltimore, MD, USA; h The Wilmer Eye Institute, Johns Hopkins University, Baltimore, MD, USA; i Department of Epidemiology, Johns Hopkins Bloomberg School of Public Health, Baltimore, MD, USA; j Zhongshan Ophthalmic Center, Sun Yat-sen University, Guangzhou, China; k Orbis International, New York, NY, USA; l Department of Ophthalmology, University of Calabar, Calabar, Nigeria; m Africa Vision Research Institute, Durban, Kwa-Zulu Natal, South Africa; n Massachusetts Eye and Ear, Harvard Ophthalmology, Harvard Medical School, Boston, MA, USA; o Department of Ophthalmology, University of Nairobi, Nairobi, Kenya; p Institute of Clinical and Scientific Ophthalmology and Acupuncture Jonas & Panda, Heidelberg, Germany; q Department of Ophthalmology, Medical Faculty Mannheim, Heidelberg University, Mannheim, Germany; r Institute of Molecular and Clinical Ophthalmology Basel, Basel, Switzerland; s National Institute for Health Research Biomedical Research Centre for Ophthalmology at Moorfields Eye Hospital NHS Foundation Trust and UCL Institute of Ophthalmology, London, UK; t College of Health Sciences, University of Abuja, Abuja, Nigeria; u Indian Institute of Public Health, Hyderabad, India; v Beijing Institute of Ophthalmology, Beijing Tongren Eye Center, Beijing Tongren Hospital, Capital Medical University, Beijing, China; w Beijing Ophthalmology and Visual Sciences Key Laboratory, Beijing, China; x Singapore Eye Research Institute, Singapore National Eye Center, Singapore; y Duke-NUS Medical School, Singapore; z Melbourne School of Population Health, The University of Melbourne, Melbourne, VIC, Australia; aa Brien Holden Vision Institute and School of Optometry and Vision Science, UNSW, Sydney, NSW, Australia

## Abstract

**Background:**

We undertook a Grand Challenges in Global Eye Health prioritisation exercise to identify the key issues that must be addressed to improve eye health in the context of an ageing population, to eliminate persistent inequities in health-care access, and to mitigate widespread resource limitations.

**Methods:**

Drawing on methods used in previous Grand Challenges studies, we used a multi-step recruitment strategy to assemble a diverse panel of individuals from a range of disciplines relevant to global eye health from all regions globally to participate in a three-round, online, Delphi-like, prioritisation process to nominate and rank challenges in global eye health. Through this process, we developed both global and regional priority lists.

**Findings:**

Between Sept 1 and Dec 12, 2019, 470 individuals complete round 1 of the process, of whom 336 completed all three rounds (round 2 between Feb 26 and March 18, 2020, and round 3 between April 2 and April 25, 2020) 156 (46%) of 336 were women, 180 (54%) were men. The proportion of participants who worked in each region ranged from 104 (31%) in sub-Saharan Africa to 21 (6%) in central Europe, eastern Europe, and in central Asia. Of 85 unique challenges identified after round 1, 16 challenges were prioritised at the global level; six focused on detection and treatment of conditions (cataract, refractive error, glaucoma, diabetic retinopathy, services for children and screening for early detection), two focused on addressing shortages in human resource capacity, five on other health service and policy factors (including strengthening policies, integration, health information systems, and budget allocation), and three on improving access to care and promoting equity.

**Interpretation:**

This list of Grand Challenges serves as a starting point for immediate action by funders to guide investment in research and innovation in eye health. It challenges researchers, clinicians, and policy makers to build collaborations to address specific challenges.

**Funding:**

The Queen Elizabeth Diamond Jubilee Trust, Moorfields Eye Charity, National Institute for Health Research Moorfields Biomedical Research Centre, Wellcome Trust, Sightsavers, The Fred Hollows Foundation, The Seva Foundation, British Council for the Prevention of Blindness, and Christian Blind Mission.

**Translations:**

For the French, Spanish, Chinese, Portuguese, Arabic and Persian translations of the abstract see Supplementary Materials section.

## Introduction

Eye health has been defined as “the state in which vision, ocular health, and functional ability are maximised, thereby contributing to overall health and wellbeing, social inclusion, and quality of life”.^1^ In 2020, an estimated 43 million people were blind, a further 295 million had moderate or severe distance vision impairment, 258 million had mild distance vision impairment, and 510 million had near vision impairment.^2^ In addition to these 1·1 billion people with current vision impairment, there are many more who require ongoing eye-care services to prevent vision loss from conditions such as diabetic retinopathy and glaucoma, to maintain correction of their refractive error, and to treat conditions that cause substantial morbidity without impairing vision, such as dry eye and conjunctivitis. Improving eye health can reduce mortality, improve quality of life, and increase productivity, as well as help to advance several Sustainable Development Goals, including poverty reduction, zero hunger, quality education, gender equality, and decent work.^3^, ^4^, ^5^

Despite substantial progress over the past few decades, much remains to be done to achieve eye health for all.^1^ For example, more than three-quarters of cases of distance vision impairment are due to cataract or uncorrected refractive error,^6^ conditions for which efficacious interventions exist but remain inaccessible to many and thus have not been effective. This differential access to good quality eye-care services creates and sustains inequity in terms of who remains vision impaired.^1^, ^2^ The next most common causes of vision impairment after cataract and refractive error are age-related macular degeneration, glaucoma, and diabetic retinopathy,^6^ all of which would benefit from the development of improved case finding and delivery of more effective and acceptable treatment options. A further issue for many countries is the low level of integration of eye care services within the broader health system.^1^, ^7^ For example, eye health rarely features in national health policy frameworks (particularly for primary care), is neglected in health workforce planning and the training of generalists, and is often not covered by general health financing mechanisms. An additional issue is the ageing of the global population, because eye health problems increase with age, the number of people in need of eye health services is set to increase in the coming decades, exceeding current resources. For example, despite reductions in the age-standardised prevalence of blindness and vision impairment, the number of people living with vision loss is projected to reach 1·8 billion by 2050.^2^ Finally, although new therapies and new digital technologies (eg, mobile eye care, and telemedicine) are being researched and developed, these remain disproportionately available in high-income countries. Thus we believe that substantial change is needed to achieve eye health for all, leaving no one behind.^1^

**Research in context**

**Evidence before this study**

We searched MEDLINE on Dec 12, 2018, and again on Sept 1, 2021, without language or date restrictions, for original articles using the following terms: (“eye” OR “blind*” OR “vis* impair*” OR “cataract” OR “glaucoma” OR “refractive error” OR “diabetic retinopathy” OR “age-related macular degeneration” OR “cornea*”) AND (“grand challenge*” OR “priorit* setting” OR “research priorit*” OR “health priorit*”). We reviewed reference lists of all eye health prioritisation processes, identified studies citing them, and asked experts in the field whether they were aware of any further processes. We found no previous Grand Challenges prioritisation exercise in eye health and seven reports of prioritisation processes to identify research priorities. In 2014, the James Lind Alliance in the UK did a survey and identified 11 questions that patients, carers, and clinicians hoped to see answered, with a strong focus on aetiology and prevention. The US National Eye Institute has done consultative strategic planning exercises, most recently in 2012–13 and 2019–21, as well as an Audacious Goals Initiative in 2012 which received more than 450 submissions and resulted in the pursuit of the goal of restoring vision through the regeneration of the retina. We also identified smaller, patient-focused exercises for specific conditions such as retinoblastoma in Canada, age-related macular degeneration in the USA, and herpes simplex keratitis and blepharospasm in the UK. In the global eye health space, we identified a prioritisation exercise that resulted from a workshop attended by 32 leading researchers in 2010 that generated priorities for global and regional blindness prevention research, including health services and access issues.

**Added value of this study**

By engaging a large and diverse group of eye health stakeholders from all world regions to answer and prioritise responses to one open-ended question, we were able to generate one global and seven regional lists of Grand Challenges in global eye health. By not restricting the type of participant or challenge, our lists were broad in scope and included condition-specific challenges and challenges related to health services and policy. Compared with previous exercises, the larger emphasis on health services and implementation challenges in our final priority list arises from the broader range of participants in our process, including participants from lower-income settings, and reflects the challenges of delivering eye health services in these settings.

**Implications of all the available evidence**

With the ageing global population, the need for eye health services will continue to increase, particularly in the context of pervasive inequity in access and resource limitations. We have developed a global list and regional lists of Grand Challenges in global eye health for immediate use by funders to guide investment in research and innovation. Policy makers, researchers, and service providers could build collaborations to address particular challenges by generating the evidence needed to achieve eye health for all. These lists align with recent World Health Assembly Resolutions on Integrated people-centred eye care and the UN Resolution on Vision.

As part of the *Lancet Global Health* Commission on Global Eye Health,^1^ we conducted a Grand Challenges in Global Eye Health prioritisation exercise to identify the key challenges that need to be addressed to improve eye health in the context of a growing and ageing population.

## Methods

### Overview and study design

Our approach was informed by previous Grand Challenges exercises, particularly that done for mental health.^8^ We used a three-round, Delphi-like, prioritisation process to nominate and rank challenges, involving participants from all regions globally, to develop global and regional lists of prioritised challenges. We intentionally made the process open-ended and did not prespecify areas of interest, intended beneficiaries, or a time frame. Our target audience to implement the priorities was broad, including policy makers, funders, researchers, patient groups, and industry. We report this process according to the relevant items in the reporting guideline for priority setting of health research (REPRISE).^9^

This study was approved by the Ethics Committee of the London School of Hygiene & Tropical Medicine (17487). All participants provided informed consent before commencing round 1. We included responses from all participants who completed round 1. Those who also completed rounds 2 and 3 were invited to join the manuscript authorship group. No reimbursement was offered to participants. Detailed methods are in appendix 7 (pp 2–4).

### Study management

The Grand Challenges in Global Eye Health study was initiated by *The Lancet Global Health* Commission on Global Eye Health.^1^ A core team coordinated the study (JR, JRE, EH, NM, and MJB) and was responsible for coding and thematic analysis. A steering group was recruited, including leaders in the fields of clinical and public health ophthalmology, eye health services delivery, policy, and research. The 23 members of the steering group (including eight women and 15 men, nine of whom were from low-income or middle-income countries and 14 from high-income countries) guided the overall process, including nomination of participants, questionnaire development, data synthesis, and reporting of results. Members of the steering group have been involved in other priority-setting processes. The process was carried out online between September, 2019, and April, 2020, using Qualtrics software (Qualtrics, 2019; Provo**,** UT, USA).

### Participant recruitment

We used a purposive sampling technique to recruit participants from all seven Global Burden of Disease (GBD) super-regions (hereafter called regions: central Europe, eastern Europe, and central Asia; high-income; Latin America and the Caribbean; north Africa and the Middle East; south Asia; southeast Asia, east Asia, and Oceania; and sub-Saharan Africa) and across the full range of disciplines relevant to global eye health (including decision makers, researchers, advocates, programme implementers, clinicians, and patient groups). We aimed to recruit at least 30 people per region and to have gender parity in participation.

We used three strategies to identify and recruit participants, with a focus on identifying members of typically under-represented groups. First, commissioners nominated potential participants, considering geographical distribution and gender parity. Second, an open invitation to participate was sent via publications, organisational newsletters, and social media channels that reach eye health practitioners in all regions.^10^ Finally, members of the steering group used their personal networks to identify organisations and individuals in regions where the target number of participants had not been met (ie, central Europe, eastern Europe, and central Asia; and North Africa and the Middle East).

### Round 1: identification of challenges

In round 1, to develop an initial list of priorities, we asked participants to answer one open-ended question: “What are the Grand Challenges in global eye health?”

A Grand Challenge was defined as a specific barrier, the removal of which would help to solve an important health problem. If successfully implemented, the intervention (or interventions) to address this Grand Challenge would have a high likelihood of feasibility for scaling up and impact.

Participants were invited to propose up to five Grand Challenges and to nominate ways in which each challenge could be addressed. Participants were encouraged to be as specific as possible. Round 1 was available in English, Chinese, French, and Spanish and ran for approximately 3 months, to enable recruitment of as many participants as possible.

In moving from round 1 to round 2, we used qualitative data analysis software (NVivo version 12.0; JR, JRE, EH, and NM) to categorise responses from round 1 into 21 subcategories, and organised them into four broad themes: eye conditions, health systems, patient-related factors, and research. Within each subcategory, we (JR, JRE, and EH) grouped similar responses and drafted a challenge to summarise the group. These challenges were reviewed for duplicates and clarity and consolidated into a draft list for round 2. This list was then reviewed by steering group members in two steps. In the first step, the original responses for each of the 21 separate categories were reviewed by at least two members (AF, DSF, EH, FK, GVSM, JCS, JBJ, MJB, NC, NM, RW, SG, and TYW) to see whether any of the original submissions had not been sufficiently captured or if there were unnecessary duplications in the proposed list. Feedback from this step resulted in further consolidation and additions. This shortened list was reviewed by six steering group members (BKS, HT, PTK, NC, SKW, and TYW), and further consolidated to a list of challenges for round 2, which was available in English, French and Spanish (all participants answering in Chinese in round 1 were able to complete subsequent rounds in English).

### Round 2: prioritisation of challenges

The consolidated list of challenges was presented in a random order to all participants and they were asked to select and rank the 20 challenges they considered the most important. For each participant, their top ranked challenge was allocated 20 points, their second ranked challenge 19 points, and so on; the remaining 65 challenges were not allocated any points. Participants were given approximately 3 weeks to complete round 2. After challenges were allocated points for each participant, the total number of points awarded to each challenge was summed, and challenges were then ranked (the challenge with the most points was given rank 1 and so on) to generate lists at the regional level (compiled from the results of respondents from each region) and global level (from all respondents). These lists were reviewed for clarity and overlapping concepts by the steering group, resulting in further amalgamation of some challenges.

### Round 3: ranking of challenges

The 40 challenges ranked highest by all participants in round 2 (in the global level list) were presented to all participants in round 3. Additionally, for each region, any challenge ranked in the top 40 by that region's participants that was not in the global list was also presented to participants from that region. Hence, between 41 and 48 challenges were presented to participants in round 3 (by region) in a random order. In round 3, participants ranked the priority of each challenge against four criteria (disease burden reduction, inequality reduction, immediacy of impact, and feasibility), which are outlined in the panel, on a four-point scale: very low (1 point), low (2 points), moderate (3 points), or high (4 points). The average score for each of these criteria was calculated for each challenge within each region and globally. For each challenge globally and within each region, we calculated the average score across all four criteria; the challenge with the highest average score was given rank 1 and so on.

Panel **Ranking criteria**

**Disease burden reduction**

To what extent would addressing this challenge reduce the overall burden of vision impairment and eye health disorders in the population?

**Inequality reduction**

To what extent would addressing this challenge reduce inequalities in the magnitude of disease or access to care for vision impairment or eye health disorders?

**Immediacy of impact**

To what extent would addressing this challenge produce immediate changes in the magnitude of disease or access to care for vision impairment or eye health disorders? (High <1 year; moderate 1–3 years; low 4–10 years; or very low >10 years)

**Feasibility**

To what extent is it practical and feasible to address this challenge (eg, in terms of resources needed, technical challenges to be overcome, and political support)?

### Final priority list of Grand Challenges

No prioritisation approach can fully account for and integrate all potential ranking considerations of participants. To arrive at the final list of priority Grand Challenges globally and for each region, we first ranked challenges using two approaches: (1) round 2 results, which identified participants’ overall priority challenges, and (2) round 3 results, which identified the priority challenges on the basis of the average scores across the four criteria.

We then integrated these two approaches by combining the ten highest-ranked challenges from each list and removing duplicates. We present the resulting list of priority challenges globally and for each region. Within each list, we highlight the five challenges that were ranked highest in round 3.

To account for any potential imbalance in the global list because of recruiting different numbers of participants from each region, we repeated the ranking process that was done after round 2, weighting for number of respondents and regional population.

To check for undue influence of participants from high-income country institutions identifying priorities for other regions in which they work but do not permanently reside, we identified the two regions with the highest proportion of non-residents and recalculated the ranks in round 3 after removing the high-income country participants and then compared the top ten ranked challenges generated by all participants for that region with the top ten challenges ranked by those participants from or permanently based in the region.

After completion of the process, when providing feedback on the final list and manuscript, we asked participants the extent to which they felt the final list of Grand Challenges was relevant to the intended stakeholders.

### Role of the funding source

The funders had no role in the study design, data collection, data analysis, data interpretation, or writing of the report. Employees of these funders participated as panellists in a personal capacity.

## Results

328 participants who were nominated for inclusion completed round 1, of whom 277 (84%) went on to also complete rounds 2 and round 3. Our open invitation for participants yielded another 142 respondents in round 1, of whom 59 (42%) completed all three rounds. Round 1 was completed between Sept 1 and Dec 12, 2019, round 2 between Feb 26 and March 18, 2020, and round 3 between April 2 and April 25, 2020. Therefore, 470 individuals overall contributed Grand Challenge ideas in round 1, of whom 336 (71%) completed all three rounds.

Of 336 participants who completed all three rounds, 156 (46%) were women. Participants came from 118 countries (appendix 7 p 1), with all seven world regions represented (table 1). Almost half of participants who completed all three rounds indicated that their main geographical work focus was either sub-Saharan Africa (104 [31%] of 336) or high-income countries (58 [17%]); these two regions were also the leading regions of the institutions where participants worked. Participants had a broad range of disciplines, and 239 (71%) had some lived experience of at least one eye health problem requiring treatment.

**Table 1:** Characteristics of participants completing round 1 and all three rounds of the exercise

	**Nominated challenges in round 1 (n=470)**	**Completed all three rounds (n=336)**
**Sex**		
Female	208 (44%)	156 (46%)
Male	262 (56%)	180 (54%)
GBD super-region of main work*		
Sub-Saharan Africa	146 (31%)	104 (31%)
High-income	74 (16%)	58 (17%)
South Asia	59 (13%)	44 (13%)
Southeast Asia, east Asia, and Oceania	75 (16%)	42 (13%)
Latin America and Caribbean	48 (10%)	35 (10%)
North Africa and Middle East	40 (9%)	32 (10%)
Central Europe, eastern Europe, and central Asia	28 (6%)	21 (6%)
GBD super-region of institution*		
High-income	177 (38%)	134 (40%)
Sub-Saharan Africa	104 (22%)	74 (22%)
South Asia	52 (11%)	37 (11%)
Latin America and Caribbean	38 (8%)	27 (8%)
North Africa and Middle East	37 (8%)	25 (7%)
Southeast Asia, east Asia, and Oceania	40 (9%)	21 (6%)
Central Europe, eastern Europe, and central Asia	22 (5%)	18 (5%)
**Main field of work**†		
Clinician or practitioner	200 (43%)	126 (38%)
Management or leadership in eye health	116 (25%)	89 (26%)
Clinical research	94 (20%)	76 (23%)
Eye health services research	90 (19%)	72 (21%)
Education	88 (19%)	68 (20%)
Epidemiology	64 (14%)	51 (15%)
Implementing agency (including non-governmental organisation)	58 (12%)	46 (14%)
Health service policy or planning (including Ministry of Health)	58 (12%)	44 (13%)
Other research (vision science, genetic)	37 (8%)	24 (7%)
Advocacy, corporate sector, or funder	31 (7%)	20 (6%)
International institutions (eg, WHO, Pan American Health Organization, International Agency for the Prevention of Blindness)	25 (5%)	16 (5%)
Patient group	9 (2%)	5 (1%)

Data are n (%). GBD=Global Burden of Disease Study.

* List of countries available in appendix 7 (p 1).

† Participants could nominate up to two fields of work, hence percentages will add up to more than 100%.

The 3545 responses from round 1 were collated and, after subcategorisation, we identified 161 individual challenges (figure). After review for duplication, this list was consolidated into a first draft list of 112 challenges and then further consolidated to a list of 85 challenges after review by the steering committee. This list of 85 challenges generated from round 1 are shown in appendix 7 (pp 5–7). During round 2, the list of challenges was further prioritised to a list of 40 challenges at the global level and a list of 41–48 challenges for each region (appendix 7 pp 15–16). Average scores of each criterion for each challenge and the ranking of challenges globally and for each region are shown in appendix 7 (pp 15–16). We found no difference in the global priorities selected in round 2 when we weighted the ranking process for the number of respondents from each region or when we weighted for the population of the region.

**Figure:**
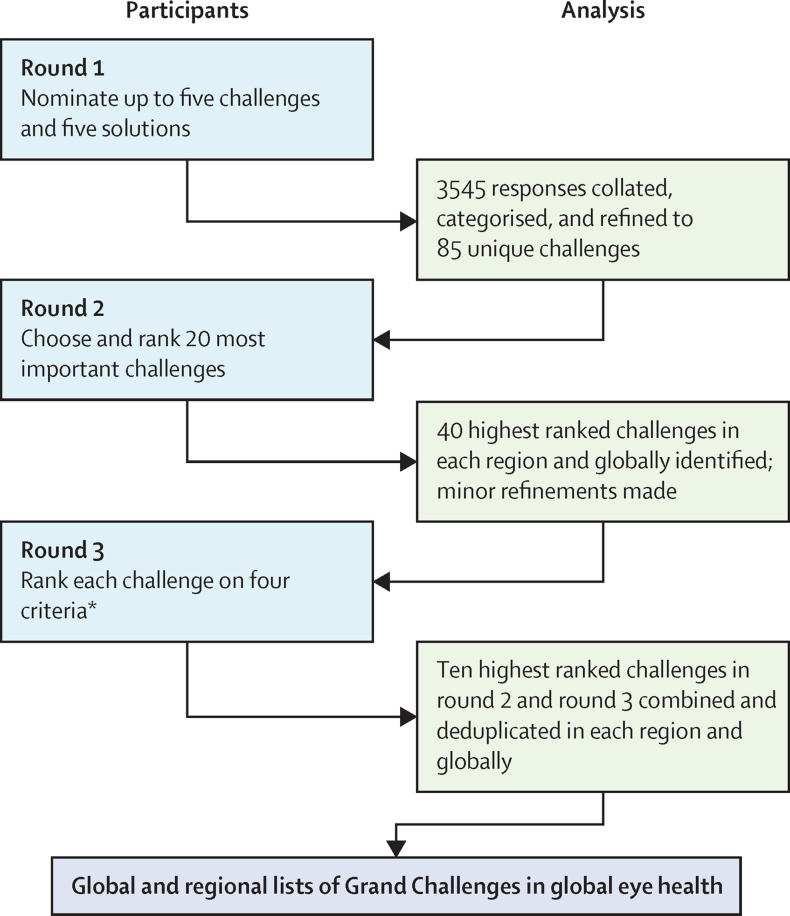
Summary of the process undertaken to identify the global and regional Grand Challenges in global eye health *Disease burden reduction, inequality reduction, immediacy of impact, and feasibility (panel).

Following this process, 16 Grand Challenges were prioritised at the global level, which we grouped into four categories (table 2). There were six challenges on detection and treatment of conditions, including cataract, refractive error, glaucoma, diabetic retinopathy, services for children, and screening for early detection. Two further challenges focused on addressing shortages in human resources and five challenges involved other health service and policy factors, including strengthening policies, integration between levels of eye care and between eye care and other health services, strengthening the health information system, and ensuring budget allocation for eye care. Finally, three of the prioritised challenges focused on improving access to care and promoting equity, including strategies to target marginalised or under-served groups, reducing out-of-pocket costs, and improving access to and uptake of services for all. The challenges for cataract, refractive error, and child eye health were the five highest prioritised challenges globally, alongside targeting marginalised groups and reducing out-of-pocket costs.

**Table 2:** Top 16 Grand Challenges in global eye health, prioritised via the Delphi method

	**Round 2 rank**	**Round 3 rank**
**Detection and treatment of conditions**		
Develop models to encourage population demand and ensure access to accurate refraction and affordable, good quality spectacles*	15	1
Identify and implement strategies to improve the quality, productivity, equity, and access of cataract services*	27	2
Improve child eye health: integrate evidence-based primary eye-care services for children into general children's health services and ensure strong connections to secondary eye-care services; develop and implement sustainable school eye health programmes, including screening and management for refractive error and amblyopia, that are well integrated within education services*	2	3
Develop and implement one-stop services for people with diabetes, through integrating diabetic retinopathy screening services with general diabetes care and developing robust systems to ensure ongoing follow-up and referral for assessment and treatment	37	8†
Develop and implement evidence-based, effective, sustainable, and context-relevant screening and early detection strategies for eye conditions	11	10
Develop and implement effective, accessible, and inexpensive pathway approaches for screening, diagnosing, monitoring, and managing glaucoma	10	21
**Health services and policy**		
Develop and implement evidence-based strategies for the effective integration of eye health services between primary level and secondary and tertiary levels, improving referral pathways; ensuring that there is recognition of those who need secondary level care and that there is a timely, reliable, accessible, and affordable mechanism connecting people to the care they need	4	7
Develop and implement evidence-based strategies for the effective integration of eye care at the primary care level and with other medical services (eg, child health, diabetes, and non-communicable diseases services); ensuring that services are widely accessible, affordable, and of high quality, meeting the primary eye care needs of the population	7	8†
Ensure financing for eye health exists within national budgets and financing structures, and increase the investment	3	13
Encourage governments to prioritise delivering integrated people-centred eye health care services for Universal Health Coverage	1	16
Strengthen the health information system for eye health within health facilities, integrating them into national systems	9	34
**Access and equity**		
Develop and implement services that are designed to prioritise reaching marginalised or vulnerable groups (eg, women, poor communities, Indigenous people, ethnic minorities, people with disabilities, people in aged care, and people in prisons and refugee camps) and people living in rural communities with quality, affordable eye services*	5	4
Develop and implement strategies that reduce out-of-pocket costs for those requiring eye care who are unable to afford full-cost services (eg, subsidy, tiered pricing, and insurance)*	25	5
Develop and implement appropriately responsive programmes to increase the access to and use of eye health services and treatment (eg, reduce barriers to accessing services and increase demand through greater awareness of need and confidence in health care provision)	8	11
**Human resource capacity**		
Increased support to geographical regions with particularly severe shortages in eye health resources, by international bodies, professional bodies and colleges, and non-governmental organisations	38	6
Strengthen leadership and public health expertise across all levels of eye health care and ensure national level leadership has the ability to influence policy and resource allocation (including strengthening regional and national professional bodies for eye health practitioners)	6	28

The rank from round 2 is from 85 challenges presented to all participants; the rank from round 3 is from 41–48 challenges presented to participants according to region.

* The top five challenges ranked by disease-burden reduction, impact on equity, immediacy of impact, and feasibility.

† Tied score.

Only four challenges were ranked in the top ten in both rounds 2 and 3, reflecting issues that are considered conceptually important and promising in terms of reducing disease burden and inequality, and having immediacy of impact and feasibility. These were the challenges relating to child eye health, integration of eye health with other health services, integration of eye health across levels of care, and incorporating equity into the design of services (table 2).

We repeated the process used for the global list to generate a final list for each region to provide more context-specific priorities (appendix 7 pp 8–14). For example, improving cataract services was the only challenge included in both the top five challenges globally and in all regions, whereas strengthening diabetic retinopathy services was ranked in the top five challenges in three regions (north Africa and the Middle East; Latin America and Caribbean; and central Europe, eastern Europe, and central Asia) but not globally.

We checked the influence of high-income country participants prioritising challenges for a region in which they work but is not where they permanently reside. The two regions with the highest proportion of non-resident participants from high-income countries were southeast Asia, east Asia, and Oceania (21 [50%] of 42 participants completing all three rounds) and sub-Saharan Africa (30 [29%] of 104). When non-resident participants from high-income countries were removed from the responses for these regions, the top ten ranked challenges differed by only two challenges in southeast Asia, east Asia, and Oceania and by only one in sub-Saharan Africa (appendix 7 p 17).

Furthermore, participants were asked the extent to which they felt the final list of Grand Challenges was relevant to a range of stakeholders. Most of the 270 participants who responded considered the list to be either extremely or fairly relevant for policy makers (258 [96%]), funders (254 [94%]), researchers (252 [93%]), and service planners or managers (250 [93%]), while fewer participants considered it of the same relevance for service providers (225 [83%]), industry (212 [79%]), or people needing eye health services (199 [74%]).

## Discussion

To our knowledge this prioritisation process is the most geographically diverse consultation to date to identify a clear set of priorities to be addressed in global eye health. We engaged 336 people from 118 countries, representing a broad range of disciplines in eye health, including clinical practice, eye health services management, research and policy making, as well as lived experience of eye health problems.

The prioritised challenges are broad ranging, and consequently addressing them calls for different responses—including advocacy, coordinated action, and research—from stakeholders including patients, policy makers, researchers, funders, programme managers, and industry. We believe more research will be required to address most of the prioritised challenges (eg, treatment for glaucoma). Other challenges can be addressed using evidence-informed advocacy, such as ensuring financing for eye health within national budgets and strengthening leadership for eye health.

We believe the lists of prioritised challenges in global eye health presented in this study serve as a starting point for immediate action by researchers and research funders. As a follow-up to *The Lancet Global Health* Commission on Global Eye Health, we aim to use these Grand Challenges as the basis for a collaborative workshop to generate a research agenda and priority research questions for global eye health, and to establish collaboration opportunities and develop a strategy for periodic monitoring of progress regionally and globally. This process would provide an opportunity for consortia, networks, advocacy organisations, universities, and governments to organise their activities around one or more of the challenges. Furthermore, we call for research funders to use the challenges to guide their research investments. Our results can also be used for other priority-setting exercises, such as that underway by the Cochrane Eyes and Vision Group.^11^

An essential initial step is to identify the existing evidence (and corresponding gaps) for each challenge through high-quality evidence synthesis. We anticipate that the level of evidence across the challenges is variable, so a range of evidence synthesis approaches will be required. For example, several systematic or scoping reviews have been done to assess cataract services in terms of access, coverage, quality, and equity,^12^, ^13^, ^14^ but not productivity. The number and quality of studies within these reviews vary greatly; a review that focused on equity was limited to low-income and middle-income countries and identified only two studies from rural China,^13^ whereas a review that focused on global quality identified 143 studies, predominantly (65%) from high-income countries.^12^ By contrast, there is a scarcity of reviews or primary studies on refractive error services or integration of eye health, both of which are areas in need of urgent attention.

Several of the disease-focused and equity-relevant challenges could be explored through research within so-called implementation laboratories, wherein health system providers and researchers collaborate to embed rigorous research methods into initiatives to improve health care in defined populations to generate generalisable knowledge on what works, for whom, and under what circumstances.^15^, ^16^ This approach might be particularly useful for cataract and refractive error services in low-income and middle-income countries, where effective service coverage rates are low.^17^, ^18^ Disparities are also evident within countries, with marginalised or under-served groups having worse access to good quality services,^19^ even in high-income countries.^20^, ^21^ Implementation research could be used to identify effective strategies to deliver the known efficacious treatments for cataract and refractive error to all who could benefit from them, including marginalised and under-served groups.

In contrast with cataract and refractive error, which have one-off treatments, glaucoma and diabetic retinopathy are chronic eye diseases that require early detection and treatment to avoid vision loss. For diabetic retinopathy, screening for early detection remains a key priority, and research into models of integrated care are needed, which could include testing the use of smartphone-based imaging and artificial intelligence-based image analysis.^22^, ^23^ Early detection for glaucoma is more complex and resource-intensive than for diabetic retinopathy, and more evidence is needed on the most effective treatment approaches, particularly in low-income and middle-income countries. Once these issues have been addressed, the focus could shift to implementation research to maximise service coverage.

Little research has been done that addresses health services and policy challenges for eye health, including integration, workforce issues, and sustainable financing.^1^, ^24^ To address this gap, research questions regarding health services and policy could be embedded within national research agenda that are aligned to eye health policies and plans.^7^, ^25^ The upcoming roll-out of the package of eye-care interventions by WHO provides an opportunity to embed research questions on integration, health services, financing, and policy into a health system strengthening process.^26^

Previous processes undertaken to generate research priorities for eye health have some alignment with our results. In 2010, the International Agency for the Prevention of Blindness convened a workshop attended by 32 leading researchers in global eye health who generated priorities for global and regional research into blindness prevention.^27^ This list has some overlap with the challenges we identified through our Delphi-like process, including health services and policy and access issues. In the UK, the James Lind Alliance undertook a prioritisation survey process in 2014 to ascertain research priorities from 2220 patients, carers, and clinicians.^28^ The final priorities list included 12 separate eye conditions. In common with our findings was the inclusion of cataract, refractive error, glaucoma, and children's eye health among the top priorities. Our challenges align with some of the research needs outlined across the seven areas of emphasis in the 2021 Strategic Plan of the US National Eye Institute, particularly the area of public health and disparities.^29^ Smaller patient-focused exercises have been done for specific conditions not included in our global list, such as retinoblastoma in Canada,^30^ age-related macular degeneration in the USA,^31^ and herpes simplex keratitis^32^ and blepharospasm^33^ in the UK.

A key strength of our process is the broad global engagement achieved, with participants from 118 (61%) of 195 countries and territories, with these regions representing 93% of the global population, and at least 20 people working in each region. Our multi-pronged recruitment strategy meant we reached a broad range of participants from all regions of the world, including from countries and disciplines not typically included in international networks and debates. This geographical diversity enabled us to extend the usual approach and generate lists of regional priorities in addition to the global list. A further extension of the usual Grand Challenges approach was retaining the top-ranking challenges after round 2 in the final prioritised list. This approach allowed us to retain those challenges considered conceptually important that perhaps do not have a direct link to disease reduction (eg, strengthening the health information system) or are not considered to have high immediacy (eg, establishing effective glaucoma treatment). Despite this approach, we recognise that the process we followed meant that rare conditions, which have a substantial impact on individuals and families, and essential elements of eye health services, such as rehabilitation services, are absent from the list.

We also recognise the limitations of our approach. First, few participants nominated “patient group” among their two main fields of work despite a high proportion indicating that they had experienced at least one eye health problem. This might reflect that the eye health problems of most participants were not severe or were being effectively managed, and the challenges prioritised might have differed had more participants with more severe eye health problems been included. Second, despite our efforts, some regions remained under-represented in our panel, which we might have overcome if we expanded the languages we used and explored and addressed other barriers to participation. Third, we acknowledge our regional lists were generated from participants who worked in each region rather than exclusively being from or permanently residing in the region. However, our analysis showed that the priorities identified by these participants were not meaningfully different from those participants from the region, confirming participants from high-income countries did not skew the perspective. Fourth, there was possible confirmation bias in the process of collating, categorising, and refining responses after round 1 and round 2. We attempted to mitigate this potential bias by having two members of the core team reviewing each coded item followed by two members of the steering group. Fifth, round 1 took place before the COVID-19 pandemic and so the new challenges to eye health arising as a result of the pandemic are missing from this list. Finally, we recognise that these processes often include a workshop to finalise the results, which was not feasible given the large number of participants involved. However, the list of prioritised challenges was shared with all participants and suggestions for implementation sought; these suggestions have been incorporated into this Article.

We have built on previous Grand Challenges exercises to engage several hundred people across all world regions. The resulting lists of global and regional priorities can be used by a broad range of stakeholders to guide investment and action to strengthen eye health services and work towards eye health for all. We believe this process has provided a framework for the high-quality research that WHO called for in its World Report on Vision.^7^ This framework can be used by countries in their pursuit of integrated people-centred eye care, as endorsed at the World Health Assembly in 2020^34^ and supported by the UN Resolution on Vision.^35^

## Data sharing

Data generated during this process are included in the manuscript and appendix.

## Declaration of interests

MJB reports grants (in support of the work of the *Lancet* Global Health Commission) from The Wellcome Trust, The Queen Elizabeth Diamond Jubilee Trust, Sightsavers, The Fred Hollows Foundation, The British Council for the Prevention of Blindness, Moorfields Eye Charity, The Seva Foundation, and Christian Blind Mission during the conduct of the study. NC reports personal fees from Belkin Vision. DSF reports personal fees from W L Gore, Bausch and Lomb, Life Biosciences, Thea, and iDx outside of the submitted work; and is a member of the Board of Orbis International. TYW reports grants from Allergan, Bayer, Boehringer Ingelheim, Eden Ophthalmic, Genentech, Iveric Bio, Merck, Novartis, Oxurion (formerly ThromboGenics), Roche, Samsung, Shanghai Henlius, Zhaoke Pharmaceutical, and Aldropika Therapeutics, and is co-founder of start-up companies Plano and EyRiS. PTK reports stockholding for Radiance Therapeutics and Optceutics; personal fees from Aerie, Alcon, AstraZeneca, Bausch + Lomb, Genetech, Novartis, Pfizer, and Sanofi-Aventis outside of the submitted work; being a board member of Moorfields Eye Hospital; and having a patent pending for Biochannel Device. HT reports leadership roles for the International Council of Ophthalmology and the Ophthalmology Foundation (unpaid roles). SKW reports board membership (unpaid) of Christian Blind Mission-USA. All other authors declare no competing interests.

## Supplementary Materials

French translation of the abstract

Spanish translation of the abstract

Chinese translation of the abstract

Portuguese translation of the abstract

Arabic translation of the abstract

Persian translation of the abstract
