# Supplementary material for: Grand Challenges in global eye health: a global prioritisation process using Delphi method
Source: Lancet Healthy Longev. 2022 Jan;3(1):e31–41. doi: 10.1016/S2666-7568(21)00302-0 (PMC8732284; doi:10.1016/S2666-7568(21)00302-0)
Supplement: French translation of the abstract [file mmc1.pdf]

THE LANCET
Healthy Longevity

Supplementary appendix 1

This translation in French was submitted by the authors and we reproduce it as supplied. It has not been peer reviewed. *The Lancet*'s editorial processes have only been applied to the original in English, which should serve as reference for this manuscript.

Supplement to: Ramke J, Evans JR, Habtamu E, et al. Grand Challenges in global eye health: a global prioritisation process using Delphi method. *Lancet Healthy Longev* 2022; **3:** e31–41.

Cette traduction en français a été proposée par les auteurs et nous l'avons reproduite telle quelle. Elle n'a pas été examinée par des pairs. Les processus éditoriaux du *Lancet* n'ont été appliqués qu'à l'original en anglais et c'est cette version qui doit servir de référence pour ce manuscrit.

Grands défis de la santé oculaire mondiale : un exercice de priorisation mondiale utilisant la méthode Delphi

Contexte

Nous avons entrepris un exercice de priorisation des Grands défis de la santé oculaire mondiale afin d'identifier les questions clés qui doivent être résolues pour améliorer la santé oculaire dans le contexte d'une population vieillissante, d’inégalités persistantes dans l'accès aux soins et de limitations généralisées des ressources.

Méthodes

En nous inspirant des méthodes utilisées lors d’études précédentes sur des Grands Défis, nous avons recouru à une stratégie de recrutement en plusieurs étapes afin de réunir un panel diversifié issu de toutes les régions du monde en vue de réaliser un processus de hiérarchisation de type Delphi en trois étapes pour désigner et classer les défis en matière de santé oculaire mondiale. Ce processus nous a permis d’élaborer des listes de priorités mondiales et régionales.

Résultats

Entre le 1er septembre et le 12 décembre 2019, 470 personnes réalisèrent le premier tour du processus, dont 336 les trois tours (deuxième tour entre le 26 février et le 18 mars 2020, troisième tour entre le 2 et le 25 avril 2020) ; 156 (46 %) parmi les 336 étaient des femmes et 180 (54 %) étaient des hommes. La part de participants qui travaillaient dans chaque région variait de 104 (31 %) en Afrique subsaharienne à 21 (6 %) en Europe centrale, en Europe de l'Est et en Asie centrale. Sur les 85 défis identifiés à l'issue du premier tour, 16 défis ont été classés par ordre de priorité au niveau mondial ; six d'entre eux portaient sur la détection et le traitement d'affections spécifiques (cataracte, vice de réfraction, glaucome, rétinopathie diabétique, prise en charge des enfants et dépistage/détection précoce), deux visaient à remédier aux pénuries de ressources humaines, cinq portaient sur d'autres facteurs liés aux services et politiques de santé (notamment le renforcement des politiques, l'intégration, les systèmes d'information sanitaire et les allocations budgétaire) et trois portaient sur l'amélioration de l'accès aux soins et la promotion de l'équité.

Interprétation

Cette liste de grands défis sert de point de départ à une action immédiate des bailleurs de fonds pour orienter les investissements dans la recherche et l'innovation en santé oculaire. Il met au défi les chercheurs, les cliniciens et les décideurs politiques d’établir des collaborations pour répondre à des enjeux précis.
