# Supplementary material for: Grand Challenges in global eye health: a global prioritisation process using Delphi method
Source: Lancet Healthy Longev. 2022 Jan;3(1):e31–41. doi: 10.1016/S2666-7568(21)00302-0 (PMC8732284; doi:10.1016/S2666-7568(21)00302-0)
Supplement: Spanish translation of the abstract [file mmc2.pdf]

THE LANCET
Healthy Longevity

## Supplementary appendix 2

This translation in Spanish was submitted by the authors and we reproduce it as supplied. It has not been peer reviewed. *The Lancet*'s editorial processes have only been applied to the original in English, which should serve as reference for this manuscript.

Supplement to: Ramke J, Evans JR, Habtamu E, et al. Grand Challenges in global eye health: a global prioritisation process using Delphi method. *Lancet Healthy Longev* 2022; **3:** e31–41.

Los autores nos proporcionaron esta traducción al español y la reproducimos tal como nos fue entregada. No la hemos revisado. Los procesos editoriales de *The Lancet* se han aplicado únicamente al original en inglés, que debe servir de referencia para este manuscrito.

## Grandes Desafíos de la salud ocular global: Un proceso de priorización global usando el método Delphi


**Antecedentes**

Realizamos un ejercicio de priorización de Grandes Desafíos en la Salud Ocular Global para identificar los problemas clave a resolver, para mejorar la salud ocular en el contexto de una población que envejece, buscando eliminar desigualdades persistentes en el acceso a la atención en salud y mitigar la limitación generalizada de recursos.

**Métodos**

Basándonos en la metodología de Grandes Desafíos previamente usada en otros estudios, seleccionamos en varios pasos a un panel diverso de individuos de un rango de disciplinas relevantes a la salud ocular, de todas las regiones del mundo para llevar a cabo un proceso de priorización en línea de tres rondas, similar al proceso de priorización Delphi para nominar y clasificar los desafíos en la salud ocular global. A través de este proceso desarrollamos listas de prioridades globales y regionales.

**Resultados**

Entre el 1 de septiembre y el 12 de diciembre de 2019, 470 individuos completaron la ronda 1 del proceso, en total, 336 personas completaron las tres rondas (ronda 2 entre el 26 de febrero y el 18 de marzo, 2020 y la ronda 3 entre el 2 y el 25 de abril, 2020) 156 (46%) de 336 fueron mujeres, 180 (54%) fueron hombres; la proporción de participantes en cada región vario de 104 (31%) en África Subsahariana a 21 (6%) en Europa Central / Este y Asia Central. De los 85 retos identificados en la ronda 1, 16 fueron priorizados a nivel mundial; 6 se centraron en detección y tratamientos específicos (cataratas, defectos de refracción, glaucoma, retinopatía diabética, servicios para niños y detección precoz / detección temprana), 2 abordaron la escasez de recursos humanos, 5 abordaron otros servicios de salud y factores de política (incluidas políticas de fortalecimiento, integración, sistemas de información en salud y asignación presupuestaria) y 3 sobre el mejoramiento del acceso a la atención y la búsqueda de la equidad.

**Interpretación**

Esta lista de Grandes Desafíos sirve como punto de partida para la acción inmediata de las entidades financiadoras, orientando la inversión en investigación e innovación en salud ocular y motivando a los investigadores, médicos y legisladores a colaborar para resolver los diferentes desafíos específicos.
