# Supplementary material for: Grand Challenges in global eye health: a global prioritisation process using Delphi method
Source: Lancet Healthy Longev. 2022 Jan;3(1):e31–41. doi: 10.1016/S2666-7568(21)00302-0 (PMC8732284; doi:10.1016/S2666-7568(21)00302-0)
Supplement: Chinese translation of the abstract [file mmc3.pdf]

THE LANCET
Healthy Longevity

Supplementary appendix 3

This translation in Chinese was submitted by the authors and we reproduce it as supplied. It has not been peer reviewed. *The Lancet*'s editorial processes have only been applied to the original in English, which should serve as reference for this manuscript.

Supplement to: Ramke J, Evans JR, Habtamu E, et al. Grand Challenges in global eye health: a global prioritisation process using Delphi method. *Lancet Healthy Longev* 2022; **3:** e31–41.

此简体中文译文由作者提交，我方按照提供的版本刊登。此译文并未经过同行审阅。医学期刊《柳叶刀》的编辑流程仅适用于英文原稿，英文原稿应作为此手稿的参考。

全球眼健康重大挑战：基于德尔菲法的全球优先级排序研究

背景

我们进行了一项全球眼健康重大挑战优先级排序研究，以确定必需解决的关键性问题，从而在人口老龄化的背景下提高眼健康水平、消除长期以来医疗服务可及性不平等问题、并且缓解普遍存在的资源匮乏问题。

方法

借鉴之前重大挑战研究中使用的方法，我们使用多轮招募参与者的策略，召集了来自全球各个地区、不同眼健康学科领域的多元化的研究小组，进行了三轮类似德尔菲法的线上优先级排序研究，请参与者提出有哪些全球眼健康领域的挑战并对其优先级进行排序。通过这种方法，制定全球性和地区性的优先级清单。

结果

2019 年 9 月 1 日至 12 月 12 日期间，470 人完成了第一轮调查，其中 336 人完成了所有三轮调查（第二轮时间为 2020 年 2 月 26 日至 3 月 18 日，第三轮时间为 2020 年 4 月 2 日至 4 月 25 日）。336 人中 156 人（46%）为女性，180 人（54%）为男性。在各个地区工作的参与者的比例从撒哈拉以南非洲的 104 人（31%）到中欧、东欧和中亚的 21 人（6%）不等。在第一轮确定的 85 项特殊挑战中，16 项挑战具有全球优先性；6 项侧重于疾病检出和治疗（白内障、屈光不正、青光眼、糖尿病视网膜病变、儿童服务和筛查/早期检测），2 项侧重于解决人力资源短缺问题，5 项侧重于其他卫生服务和政策因素（包括加强政策、整合、卫生信息系统和预算分配），3 项关注改善服务可及性和促进公平。

解读

重大挑战清单是资助者立即采取行动指导眼健康研究和创新投资的基点。同时也能促进研究人员、临床医生和决策者开展合作以应对特定挑战。
