# Supplementary material for: Grand Challenges in global eye health: a global prioritisation process using Delphi method
Source: Lancet Healthy Longev. 2022 Jan;3(1):e31–41. doi: 10.1016/S2666-7568(21)00302-0 (PMC8732284; doi:10.1016/S2666-7568(21)00302-0)
Supplement: Portuguese translation of the abstract [file mmc4.pdf]

THE LANCET
Healthy Longevity

Supplementary appendix 4

This translation in Portuguese was submitted by the authors and we reproduce it as supplied. It has not been peer reviewed. The Lancet's editorial processes have only been applied to the original in English, which should serve as reference for this manuscript.

Supplement to: Ramke J, Evans JR, Habtamu E, et al. Grand Challenges in global eye health: a global prioritisation process using Delphi method. *Lancet Healthy Longev* 2022; **3:** e31–41.

Esta tradução em português foi submetida pelos autores e nós não fizemos quaisquer alterações. Esta versão não foi revista por pares. O processo editorial do *The Lancet* só foi aplicado à versão original em inglês, que deve servir como referência para este artigo.

Grandes Desafios da Saúde Ocular Global: processo de priorização global utilizando o método de Dephi

Enquadramento
Realizámos um exercício de definição de prioridades sobre os Grandes Desafios da Saúde Ocular Global para identificar as questões-chave que devem ser tidas em conta para melhorar a saúde ocular no contexto de uma população em envelhecimento, para eliminar desigualdades persistentes no acesso à saúde e para minimizar limitações generalizadas de recursos.

Métodos
Baseando-nos em métodos utilizados em estudos anteriores do tipo Grandes desafios, usámos uma estratégia de recrutamento com várias etapas por forma a reunir um painel diversificado de indivíduos, provenientes de todas as regiões do mundo e de diferentes disciplinas relevantes para a saúde ocular global, para realizar um processo de priorização, online de três rondas do tipo Delphi, para identificar e classificar os desafios na saúde ocular global. Através deste processo desenvolvemos listas de prioridades tanto globais como regionais.

Resultados

Entre 1 de setembro e 12 de dezembro 2019, 470 indivíduos completaram a primeira ronda, entre os quais 336 pessoas completaram as três rondas (a ronda 2 decorreu entre 26 de fevereiro e 18 de março, 2020 e a ronda 3 decorreu entre 2 de abril e 25 abril, 2020) 156 (46%) mulheres de um total de 336, 180 (54%) homens. A proporção de indivíduos proveniente de cada região variou desde 104 (31%) provenientes da África Subsaariana a 21 (6%) provenientes da Europa Central / Oriental e Ásia Central. De 85 desafios identificados após a ronda 1, 16 foram considerados prioritários a nível global; seis concentraram-se na deteção e tratamentos de condições de saúde (catarata, erro refrativo, glaucoma, retinopatia diabética, serviços para crianças e triagem / detecção precoce), dois concentraram-se em abordar a escassez de recursos humanos, cinco noutros serviços de saúde e fatores políticos (incluindo o reforço de políticas, integração, sistemas de informação em saúde e afetação orçamental) e três na melhoria do acesso aos cuidados e promoção da equidade.

Interpretação
Esta lista dos Grandes desafios serve como um ponto de partida para ações imediatas dos financiadores com vista a orientar o investimento em investigação e inovação em saúde ocular. Desafia os investigadores, clínicos e decisores políticos a construírem colaborações para enfrentar desafios específicos.
