# Supplementary material for: Grand Challenges in global eye health: a global prioritisation process using Delphi method
Source: Lancet Healthy Longev. 2022 Jan;3(1):e31–41. doi: 10.1016/S2666-7568(21)00302-0 (PMC8732284; doi:10.1016/S2666-7568(21)00302-0)
Supplement: Arabic translation of the abstract [file mmc5.pdf]

THE LANCET
Healthy Longevity

Supplementary appendix 5

This translation in Arabic was submitted by the authors and we reproduce it as supplied. It has not been peer reviewed. *The Lancet*'s editorial processes have only been applied to the original in English, which should serve as reference for this manuscript.

Supplement to: Ramke J, Evans JR, Habtamu E, et al. Grand Challenges in global eye health: a global prioritisation process using Delphi method. *Lancet Healthy Longev* 2022; **3:** e31–41.

تم تقديم هذه الترجمة باللغة العربية من قبل المؤلفين ونعيد إنتاجها كما هو مُقدم. إنها لم تخضع لاستعراض الأقران. تم تطبيق عمليات تحرير لانسيت فقط على النص الأصلي باللغة الإنجليزية، والذي يجب أن يكون بمثابة مرجع لهذه المخطوطة.

التحديات الكبرىٰ في صحة العين في العالم، عملية تحديد الأولويات في العالم باستخدام طريقة دلفي

الخلفية:
لقد اجرينا تمرين تحديد الاولويات للتحديات الكبرىٰ في صحة العين في العالم و ذلك لمعرفة القضايا الأساسية التي يجب التوجه اليها لتحسين صحة العين لدىٰ كبارالسن والحد من التمييز المستمر في الحصول علىٰ الرعاية الصحية و تخفيف العوائق السائدة للموارد.
الاساليب:
مع استخدام الأساليب المعتمدة في دراسات التحديات الكبرىٰ السابقة، تم استعمال استراتيجية توظيف متعددة الخطوات لتكوين لجنة متنوعة من أشخاص ذوي تخصصات ذات صلة بصحة العين في شتى أرجاء العالم للمشاركة في عملية تحديد الأولويات مكونة من ثلاث مراحل مشابهة لدلفي عبر الانترنت و ذلك لتعيين و ترتيب التحديات في مجال صحة العين في العالم. و لقد تم وضع قوائم الاولويات العالمية و الاقليمية من خلال هذه العملية.
النتائج:
في الفترة الزمينة ما بين الاول من ايلول و 12 كانون الاول عام 2019، اكمل 470 شخصاً المرحلة الاولىٰ من العملية. و انهى 336 منهم كافة العمليات الثلاث (اجريت المرحلة الثانية بين 26 شباط و 18 آذار عام 2020 و المرحلة الثالثة بين 2 نيسان و 25 نيسان من العام نفسه)و كان 156 (46 %) من مجموع 336 شخصاً امرأة و 180 (54 %) رجلاً. وتراوحت نسبة المشاركين في كل منطقة من 104 أشخاص (31 %) من جنوب الصحراء الكبرىٰ في افريقيا الىٰ 21 (6 %) شخصاً من وسط و شرق اوروبا و آسيا الوسطىٰ. تم تحديد اولويات 16 تحدياً علىٰ المستوىٰ العالمي من مجموع 85 تحدياً فريداً تم تعيينها بعد المرحلة الاولىٰ. تركزت ستة منها علىٰ تشخيص الحالات و علاجها (الساد و الأخطاء الانكسارية و الزرق و اعتلال الشبكية السكري و خدمات الأطفال و الغربلة / للتشخيص المبكر) واثنتان منها علىٰ قلة الموارد الانسانية و خمسة منها علىٰ الخدمات الصحية الاخرىٰ و عوامل خطة العمل (بما فيها تعزيز خطط العمل و التكامل و أنظمة المعلومات الصحية و تخصيص الميزانية) و ثلاثة منها علىٰ تحسين الوصول الىٰ الرعاية الصحية و تعزيز المساواة.
التحليل:
تعمل هذه القائمة للتحديات الكبرىٰ كنقطة انطلاق لاتخاذ إجراءات فورية من قبل الممولين وذلك لتوجيه الاستثمار في البحث و الابتكار في مجال صحة العين و تتحدىٰ الباحثين والأطباء وواضعي السياسات للتعاون في مواجهة التحديات الخاصة.
