# Supplementary material for: Grand Challenges in global eye health: a global prioritisation process using Delphi method
Source: Lancet Healthy Longev. 2022 Jan;3(1):e31–41. doi: 10.1016/S2666-7568(21)00302-0 (PMC8732284; doi:10.1016/S2666-7568(21)00302-0)
Supplement: Persian translation of the abstract [file mmc6.pdf]

THE LANCET
Healthy Longevity

Supplementary appendix 6

This translation in Persian was submitted by the authors and we reproduce it as supplied. It has not been peer reviewed. The Lancet's editorial processes have only been applied to the original in English, which should serve as reference for this manuscript.

Supplement to: Ramke J, Evans JR, Habtamu E, et al. Grand Challenges in global eye health: a global prioritisation process using Delphi method. *Lancet Healthy Longev* 2022; **3:** e31–41.

این ترجمه به زبان فارسی توسط نویسندگان ارسال شده است و ما آن را همانطور که تهیه می شود بازتولید می کنیم. مورد
فقط در اصل به زبان انگلیسی اعمال شده است ، که باید به عنوان Lancet بررسی قرار نگرفته است. مراحل تحریریه
مرجع برای این نسخه خطی باشد.

چالش های جدی (بزرگ) در سلامت چشم جهانی: یک فرآیند اولویت بندی جهانی با استفاده از روش دلفی

زمینه

یک تمرین اولویت‌بندی چالش‌های جدی (بزرگ) در سلامت چشم جهانی را انجام دادیم تا مسائل کلیدی که می بایست در جهت بهبود سلامت چشم در بستر سال‌خوردگی جمعیت، اجتناب از نابرابری‌های مداوم در دسترسی به مراقبت‌های بهداشتی و کاهش محدودیت‌های گسترده منابع مورد توجه قرار گیرند را شناسایی نماییم.

روش پژوهش

با تکیه بر روش‌های مورد استفاده در مطالعات قبلی چالش‌های جدی، از استراتژی بکارگیری چند مرحله‌ای برای فراهم نمودن هیئتی متنوع از طیف گسترده ای از افراد مرتبط با سلامت چشم جهانی از کلیه مناطق جهان جهت شرکت در یک فرآیند اولویت‌بندی دلفی-مانند آنلاین سه مرحله ای و با هدف معرفی و رتبه‌بندی چالش‌ها در زمینه سلامت چشم جهانی، استفاده کردیم. از طریق این روند، لیست های اولویت جهانی و منطقه ای را تهیه نمودیم.

یافته ها

بین اول سپتامبر و 12 دسامبر 2019، 470 نفر مرحله اول روند را تکمیل نمودند و 336 نفر از آنها هر سه مرحله را به اتمام رساندند (مرحله دوم بین 26 فوریه و 18 مارس 2020 و مرحله سوم بین دوم آوریل و 25 آوریل 2020) ؛ 156 نفر از 336 نفر (46%) زن و 180 نفر (54%) مرد بودند. نسبت شرکت کنندگانی که در هر منطقه فعالیت می کردند از 104 نفر (31%) در منطقه جنوب صحرای آفریقا تا 21 نفر (6%) در اروپای مرکزی، اروپای شرقی و در آسیای مرکزی متفاوت بود. از 85 چالشی که بعد از مرحله اول شناسایی شدند، 16 چالش در سطح جهانی در اولویت قرار گرفت؛ شش مورد بر شناسایی و درمان بیماریها (آب مروارید، عیب انکساری، گلوکوم، رتینوپاتی دیابتی، خدمات برای کودکان و غربالگری/شناسایی زودهنگام)، دو مورد بر رسیدگی به کمبود منابع انسانی، پنج مورد بر سایر خدمات بهداشتی و عوامل سیاستی (از جمله تقویت سیاست ها، ادغام، سیستم های اطلاعات سلامت و تخصیص بودجه) و سه مورد بر بهبود دسترسی به مراقبت و ارتقای برابری متمرکز بود.

تفسیر

این فهرست چالشهای جدی (بزرگ) به عنوان نقطه شروعی جهت اقدام فوری توسط سرمایه گذاران برای هدایت سرمایه گذاری در تحقیق و نوآوری در زمینه سلامت چشم بکار گرفته می شود. فهرست مزبور، محققان، پزشکان و سیاستگذاران را برای ایجاد همکاریها در جهت رسیدگی به چالش های خاص به چالش می کشد.
