# Supplementary material for: Grand Challenges in global eye health: a global prioritisation process using Delphi method
Source: Lancet Healthy Longev. 2022 Jan;3(1):e31–41. doi: 10.1016/S2666-7568(21)00302-0 (PMC8732284; doi:10.1016/S2666-7568(21)00302-0)
Supplement: Supplementary appendix [file mmc7.pdf]

THE LANCET
Healthy Longevity

Supplementary appendix 7

This appendix formed part of the original submission and has been peer reviewed. We post it as supplied by the authors.

Supplement to: Ramke J, Evans JR, Habtamu E, et al. Grand Challenges in global eye health: a global prioritisation process using Delphi method. *Lancet Healthy Longev* 2022; **3:** e31–41.

Appendix 1

List 1: Countries of panellists (n=118)

Central Europe, Eastern Europe & Central Asia: Armenia, Bosnia and Herzegovina, Croatia, Czech Republic, Hungary, Kyrgyzstan, Latvia, Mongolia, Poland, Republic of Moldova, Romania, Russian Federation, Serbia, Ukraine, Uzbekistan

High-income countries: Australia, Belgium, Canada, Chile, Denmark, France, Germany, Greece, Ireland, Israel, Italy, Japan, Netherlands, New Zealand, Portugal, Singapore, Spain, South Korea, Switzerland, United Kingdom, United States of America, Uruguay

Latin-America: Antigua and Barbuda, Argentina, Bolivia, Brazil, Colombia, Costa Rica, Ecuador, Guatemala, Guyana, Haiti, Jamaica, Mexico, Paraguay, Peru, Saint Lucia, Trinidad & Tobago, Venezuela

North Africa & Middle East: Afghanistan, Bahrain, Egypt, Iran, Kuwait, Lebanon, Morocco, Oman, Palestine, Sudan, Syrian Arab Republic, Tunisia, Turkey, United Arab Emirates

South Asia: Bangladesh, Bhutan, India, Nepal, Pakistan

Southeast Asia & East Asia: Cambodia, China, Fiji, Hong Kong (S.A.R.), Indonesia, Lao, Malaysia, Papua New Guinea, Philippines, Sri Lanka, Taiwan, Thailand, Timor-Leste, Vanuatu, Viet Nam

Sub-Saharan Africa: Benin, Botswana, Burkina Faso, Burundi, Cameroon, Chad, Congo, Côte d´Ivoire, Democratic Republic of Congo, Ethiopia, Ghana, Guinea, Kenya, Liberia, Madagascar, Malawi, Mali, Mozambique, Niger, Nigeria, Rwanda, Senegal, Sierra Leone, South Africa, Tanzania, The Gambia, Togo, Uganda, Zambia, Zimbabwe

Full description of methods

Overview

Our approach was informed by previous Grand Challenges exercises, particularly that undertaken for mental health.[1] We used a three-round, Delphi-like prioritisation process to nominate and rank challenges, involving participants from all world regions to develop both global and regional priority lists (Figure 1). We intentionally made the process open-ended and did not pre-specify areas of interest (e.g. public health, health services research, clinical research, basic science), intended beneficiaries, or the time frame. Our target audience was broad, including policy makers, funders, researchers, patient groups and industry. We report this process according to the relevant items in the reporting guideline for priority setting of health research (REPRISE).[2]

Ethics approval was granted from the Ethics Committee of the London School of Hygiene & Tropical Medicine (reference number: 17487). We included responses from all participants who completed a Round 1 form. Those who also completed the second and third rounds were invited to be named a contributor within a manuscript authorship group. No reimbursement was offered to participants.

Study Management

The *Grand Challenges in Global Eye Health* was initiated by the *Lancet Global Health* Commission on Global Eye Health, and was coordinated by the International Centre for Eye Health at the London School of Hygiene & Tropical Medicine (LSHTM).[3] The day-to-day functioning of the process was coordinated by a core team at LSHTM [JR, JE, EH, NM, MB] with administrative support. This group was also responsible for the coding and thematic analysis between Rounds 1 and 2, for which they drew on assistance from qualitative researchers.

A Steering Group was drawn from among Commissioners. This included leaders in the fields of clinical and public health ophthalmology, as well as eye health services delivery, policy and research. The 23 members of the Steering Group (8 women, 9 low- or middle-income country) guided the overall scientific process, including nomination of participants, questionnaire development, data synthesis and reporting of results. Members of the Steering Group have been involved in other priority-setting processes including the James Lind Alliance Priority Setting Partnership in Sight Loss and Vision and a process convened by the International Agency for the Prevention of Blindness (IAPB) focussed on low- and middle-income countries.[4, 5] The project was also supported by other Commissioners of the *Lancet Global Health* Commission on Global Eye Health, a group of 73 leaders in eye health from 25 countries. Commissioners were invited to help identify potential participants and to take part in the process.

The process was carried out online between September 2019 and April 2020 using Qualtrics software (Qualtrics, 2019; Utah, USA, available at https://www.qualtrics.com). Prior to all rounds, Steering Group members trialled the online form for clarity and user experience, and modifications were made as required.

Participant Recruitment

We employed a purposive sampling technique to recruit participants from all seven Global Burden of Disease (GBD) super-regions (hereafter called regions: Central Europe, Eastern Europe & Central Asia; High-income countries; Latin America & Caribbean; North Africa & Middle East; South Asia; Southeast Asia, East Asia & Oceania; Sub-Saharan Africa) and across the full range of disciplines relevant to global eye health (including decision-makers, researchers, advocates, programme implementers, clinicians and patient groups). We aimed to recruit at least 30 people from each of the regions and to achieve gender-parity in participation.

We used four strategies to identify and recruit participants, with a focus on identifying members of typically under-represented groups. These strategies included:

1. Calling on the Commissioners to nominate potential participants, highlighting targets for broad geographic distribution and gender-parity;
2. Sharing the invitation to participate in publications and social media channels that reach eye health practitioners in low- and middle-income countries[6];
3. Requesting organisations, including the World Council of Optometry, International Council of Ophthalmology, Association for Research in Vision and Ophthalmology, and the International Agency for the Prevention of Blindness, to share the invitation with their members in newsletters and on social media;

4. Drawing on personal networks of the Steering Group to identify organisations and individuals in the regions where our target number of participants (30) had not been met by these other strategies (Central Europe, Eastern Europe & Central Asia and North Africa & the Middle East).

Round 1: Identification of challenges

In Round 1, to develop an initial list of priorities, we asked participants to answer one open-ended question:

> ***What are the grand challenges in Global Eye Health?***
>
> **A grand challenge was defined as** *a specific barrier that, if removed, would help to solve an important health problem. If successfully implemented, the intervention(s) to address this grand challenge would have a high likelihood of feasibility for scaling up and impact.*

Participants were invited to propose up to five grand challenges. Space was also provided to nominate ways in which each challenge could be addressed. Participants were encouraged to be as specific as possible. Round 1 was available in English, Chinese, French and Spanish.

In moving from Round 1 to Round 2, using NVivo 12.0, we (JR, JE, EH, NM) categorised and collated the responses from Round 1 into 21 categories, organised into four broad themes:

- ***Eye conditions:*** blindness and vision impairment, low vision/rehabilitation, cataract, refractive error, glaucoma, AMD, diabetic retinopathy, cornea, childhood vision loss, other;
- ***Health Systems:*** governance/leadership, human resources, financing, health information systems, service delivery, infrastructure/equipment;
- ***Patient-related factors:*** awareness, equity, access, determinants of eye health;
- ***Research***.

Within each of the 21 categories, we (JR, JE, EH) grouped similar suggestions and drafted a summary challenge that best reflected the grouped suggestions. Sometimes we retained the exact text of a submission within the group, but often we edited for clarity. When we combined the challenges across the 21 categories, we had 161 challenges. These were reviewed by JR, JE and MB for duplicates and clarity, and consolidated to 112 challenges as the draft list for Round 2.

This list was then reviewed by Steering Group members in two steps. In the first step, the original responses for each of the 21 separate categories were reviewed by at least two members (AF, DF, EH, FK, GVM, JCS, JJ, MB, NC, NM, RW, SG, TW) to see whether any of the original submissions had not been sufficiently captured or if there were unnecessary duplications in the proposed list. Feedback from this step resulted in further consolidation and additions, resulting in a list of 100. This shortened list was reviewed by six Steering Group members (BKS, HRT, PTK, NC, SW, TW), and further consolidated to a list of 85 challenges for Round 2, which was available in English, French and Spanish (all participants answering in Chinese in Round 1 were able to complete subsequent rounds in English).

Round 2: Prioritization of challenges

The list of 85 challenges was presented to all participants in a random order and they were asked to select and rank the 20 challenges they considered the most important. For each participant, their top challenge was allocated 20 points, their 2nd ranked challenge 19 points and so on; the remaining 65 challenges (not in the top 20) were allocated no points. The total number of points awarded to each challenge was summed, and then they were ordered to generate lists at the regional level (being all responses of people located in each region) and global level (being all responses). These lists were reviewed by the Steering Group (AF, BKS, DF, EH, HF, HRT, JCS, JJ, MY, NC, NM, NW, RW, SR, SW), resulting in further amalgamation of some challenges.

Round 3: Ranking of Challenges

The 40 challenges ranked highest by participants in Round 2 (the 'global list') were presented to all participants in Round 3. In addition, within each region, any challenge ranked in the top 40 by that region's participants that was not in the global list was also presented to participants from that region in Round 3. For example, the challenge of reducing the environmental impact of eye care ranked in the top 40 in only one region—Southeast Asia, East Asia & Oceania, so it was only presented to participants from this region in Round 3. In this way, between 41 and 48 challenges were presented to participants in Round 3 (depending on

their region) in a random order. In Round 3 participants were asked to rank each of the challenges against the four criteria outlined in Table 1 on a four-point scale: very low (1), low (2), moderate (3), or high (4). The average score for each of these criteria was calculated for each challenge within each region and globally. For each challenge globally and within each region we calculated the average score across all four criteria.

Final priority list

We recognised that no prioritisation approach can fully take account of and integrate all potential ranking considerations. To arrive at the final list of priority Grand Challenges globally and within each region, we first ranked the challenges using two approaches:

1. the results from Round 2, which identified participants' overall priorities (likely shaped by combining a very wide range of considerations and criteria); and
2. the average score of the four criteria in Round 3.

We then integrated these two approaches, by combining the 10 highest-ranked challenges from each of these lists and removing duplicates. The resulting list of priority challenges is presented globally and for each region. Within each list, we highlighted the five challenges that were ranked highest in Round 3.

To account for any potential imbalance in the global list due to recruiting different numbers of participants from each region, we reran the ranking process between Round 2 and Round 3 weighting for 1) number of respondents and 2) population of the region.

To check for undue influence of participants from high-income country institutions identifying priorities for other regions in which they work, we recalculated the ranks in Round 3 after removing the high-income country participants and compared the Top 10 ranked priorities generated by the whole panel for that region and the panel of participants from / permanently based in the region.

Role of the funding source

The funders of the *Lancet Global Health* Commission on Global Eye Health (listed elsewhere in this manuscript) had no role in the study design, collection, analysis or interpretation of data for this process. Employees of these funders may have participated as panellists.

References

1. Collins PY, Patel V, Joestl SS, March D, Insel TR, Daar AS, et al. Grand challenges in global mental health. *Nature* 2011;475:27-30.
2. Tong A, Synnot A, Crowe S, Hill S, Matus A, Scholes-Robertson N, et al. Reporting guideline for priority setting of health research (REPRISE). *BMC Medical Research Methodology* 2019;19:1-11.
3. Burton M, Ramke J, Marques AP, Bourne R, Congdon N, Jones I, et al. Lancet Global Health Commission on Global Eye Health: Vision Beyond 2020. *The Lancet Global Health* 2021;9:e489-e551.
4. Rowe F, Wormald R, Cable R, Acton M, Bonstein K, Bowen M, et al. The Sight Loss and Vision Priority Setting Partnership (SLV-PSP): overview and results of the research prioritisation survey process. *BMJ open* 2014;4:e004905.
5. International Agency for the Prevention of Blindness. Workshop Report: A VISION 2020 Workshop on Research for Global Blindness Prevention. International Agency for the Prevention of Blindness; 2010.
6. Ramke J, Faal H, Burton M. Take part in the Grand Challenges in Global Eye Health study. *Community eye health* 2019;32:36.

Supplementary Table 1: 85 Challenges identified during Round 1

(Arranged according to categories)

Challenge	Category
1. **Build a stronger advocacy case for investing in eye health and communicate this more effectively.** Through generating robust data on the importance of eye health in relation to sustainable development, quality of life, health in general, economics and the rapidly growing need for services. Work in a more coordinated and effective manner to communicate with governments, industry, philanthropists, donors, and the community about the importance of eye health.	Advocacy/Policy
2. Encourage governments to prioritise delivering integrated patient-centred **eye health care services for Universal Health Coverage**. [Through including a National Eye Health strategy in national health plans, policies and allocation of resources, developing evidence-based patient-centred national guidelines and protocols, governments actively overseeing the eye healthcare provider market to meet the needs of the population.]	
3. Establish infrastructure to **promote shared learning and exchange** (South-South, South-North) within the global eye health community, ensuring appropriate clinical governance and registration frameworks are enforced to ensure patient safety.	
4. **Enable participation of people and communities with eye health problems** in policy, practice and research development.	
5. Develop updated short- and long-term global, regional and national **strategies to follow *Vision 2020***.	
6. Establish and implement **minimum standards for eye health care** for use around the world.	
7. Develop further effective prevention and long-lasting, low-cost treatment approaches for **wet and dry AMD**.	AMD
8. Strengthen **planning** by national and regional governments for **eye health human resource development**. [Integrate/align eye care workforce planning with the general health system needs and policies, and draw on global technical documents]	Capacity/ human resources
9. Increased support to geographical regions with particularly severe eye health human resource shortages, by international bodies, professional bodies/colleges, NGOs .	
10. Strengthen **leadership and public health expertise** across all levels of eye health care, and ensure national level leadership has the ability to influence policy and resource allocation. Strengthen regional and national professional bodies for eye health practitioners, to contribute this process. [To set and monitor standards for training and clinical practice, and promote and provide ongoing education / professional development.]	
11. Develop **competency-based training**, responsive to local needs, including assessment and audit approaches to identify gaps and training needs of ophthalmic personnel and all relevant non-eye health care personnel to delineate roles and responsibilities of different cadres.	
12. **Train more people** in general, public health and sub-speciality ophthalmology and other cadres for the eye health workforce, particularly in regions where numbers of practitioners are limited. Strengthen and expand eye health training through online learning.	
13. Implement policies to **recruit, retain and motivate ophthalmic personnel** to deliver more productive services where they are most needed, including in the public sector or hard to reach communities e.g. through continuing professional development and transparent and participatory leadership and career progression plans at all levels.	
14. Foster **service development networks** [regional, national and international] between different eye health worker cadres, others working in related fields to help to promote the development of systems change and shared learning.	
15. Implement a **team-based service delivery model** with clearly set roles and tasks across cadres, and improve the integration of optometrists, refractionists and other allied ophthalmic personnel.	
16. Identify and implement strategies to improve the **quality, productivity, equity and access of cataract services**. E.g. accurate biometry and an appropriate range of intraocular lens power choices.	Cataract
17. Develop and scale-up training strategies that more efficiently establish and maintain **optimal skills for good quality cataract surgery** and improve patient safety, particularly through the application of **surgical simulation methods**.	
18. Develop evidence-based, integrated services to effectively detect, treat and follow-up of **treatable and preventable eye conditions in children** such as cataract, glaucoma and retinoblastoma.	Children
19. Strengthen the provision of **retinopathy of prematurity** (ROP) services at country level, for prevention, screening, treatment, follow-up and rehabilitation.	
20. Create effective and sustainable networks of **eye banks** within each country, including donor supply and corneal transplant services.	Cornea
21. Reduce sight loss from **corneal infection**, through promoting prevention and early detection strategies; strengthen the ability of ophthalmic and general health workers to identify and appropriately manage eye infections; make microbiological diagnosis and appropriate treatments more widely available.	
22. Develop new treatments to rehabilitate vision from corneal blindness for example developing tissue-engineered corneas for transplantation (**artificial corneas**) and enable **corneal limbal regeneration.**	
23. Strengthen the **health information system** (HIS) for eye health within health facilities, integrating them into national systems. [More comprehensive data collection, electronic records, integration with district and national HIS, internet connection, better data quality, increased use in monitoring and planning at the local and national level]	Data / information
24. Develop an international **standard core indicator set for eye health and eye care services**, which can be used within a national statistics register and inter-country comparisons.	
25. Strengthen **monitoring, audit and feedback on cataract** and other surgical services to improve quality, including better use of technology; and inclusion in national reporting.	
26. Develop and implement "one-stop" **services for people with diabetes**, through integrating diabetic retinopathy screening services with general diabetes care, and developing robust systems to ensure ongoing follow-up and referral for diabetic retinopathy assessment and treatment as appropriate.	Diabetes
27. Strengthen dedicated services for **young people with diabetes**, including screening and treatment for diabetic retinopathy, and adequate planning to transition care into the adult stream at an appropriate time.	
28. Develop more effective, longer-lasting, safer and affordable **treatments for diabetic retinopathy** to halt progression and preserve vision (e.g. anti-VEGF therapies).	

29. Develop population-specific **risk calculators** to provide risk predictions for the development and progression of specific eye conditions and guide the provision of screening, monitoring and treatment services (e.g. diabetic retinopathy, glaucoma).	Diagnosis
30. Identify and implement evidenced-based, effective, sustainable and context-relevant **screening / early detection strategies** for eye conditions.	
31. Develop high-quality **economic analyses for eye health** and improve the precision of estimates through global data collection collaborations. In order to make the case for increased resource allocation and to guide decision making to maximise the benefit ("Best Buys").	Economics
32. Increase **understanding of the association** between vision impairment and quality of life, aging, dementia, depression, functional decline and mortality and the impact of treatment and vision rehabilitation on these outcomes.	Epidemiology
33. Understand **modifiable biological, environmental and social determinants** of eye disease across the life course, with an emphasis on leading causes of sight loss (cataract / refractive error / glaucoma / AMD / diabetic retinopathy / corneal disease). To support the development of approaches to prevent and slow progression of disease, and addressing poverty, literacy/education, living standards, access to clean air and water and sanitation.	
34. Develop a more complete understanding of the **epidemiology of eye disease** (prevalence, distribution, incidence, risk factors), including less common conditions, bearing in mind that about a quarter of people with blindness and visual impairment are classified as "Other" – e.g. Uveitis, corneal infection, eye cancers, non-AMD retinal conditions.	
35. Ensure all eye care services are **gender-responsive**.<br>[Shaped by relevant evidence of the distribution of need and through the development of contextually appropriate strategies to deliver equitable services]	Equity / finance
36. Develop and implement services that prioritise, by design, **reaching marginalised or vulnerable groups** (poor communities, Indigenous people, ethnic minorities, people with disabilities, people in aged care, prisons, refugee camps) and **people living in rural communities** with quality, affordable eye services.	
37. Develop and rigorously test strategies that **reduce out of pocket costs** for those requiring eye care who are unable to afford full-cost services e.g. subsidy, tiered pricing, insurance. For example, by increasing the proportion of the population covered by a health insurance package that includes essential eye care.	
38. Ensure **health insurance boards** develop guidelines and governance structures on how **costs of cataract and other services can be contained** while providing good quality, affordable, equitable care.	
39. Disaggregate all eye health data to **monitor inequality** between social groups (e.g. sex/gender, place of residence, socioeconomic status) and use these data to set targets to address inequities.	
40. Ensure **financing for eye health** exists within national budgets / financing structures and increase the investment.	Finance
41. Establish '**strategic purchasing**' of eye health services by government or other payers that promotes quality and equity and maintains cost control	
42. Distribute **health spending** in line with the relative magnitude of the problem, and the benefit / cost-benefit.	
43. Develop and implement effective, accessible and inexpensive pathway approaches for **screening, diagnosing, monitoring and managing glaucoma**. This would include a **safe and simple surgical or laser procedure for glaucoma** that can be performed quickly at low cost that reduces the pressure in eyes for a sustained period without intensive ongoing care.	Glaucoma
44. Develop technology to cost-effectively and simply **monitor low risk glaucoma patients** and glaucoma suspects who do not need ophthalmologist care.	
45. Develop and implement **macrofilaricidal agents for onchocerciasis** control programmes [a one off treatment to kill the adult worms]	Onchocerciasis
46. Strengthen and complete the global **onchocerciasis elimination** effort.	
47. Identify and implement evidenced-based, effective, sustainable and context-relevant **health promotion / disease prevention** strategies for eye health, that address underlying risk factors and lead to **Healthy Ageing**.	Prevention
48. Research into the impact of **screen time and smart devices** on eye health.	
49. Develop and implement appropriately responsive **programmes to increase the use of eye health services and treatment**. Through reducing barriers to accessing services and increasing demand through greater awareness of need and confidence in the health care provision.<br>[Approaches could include co-production with patients and community groups, mobile health "m-health" solutions, social marketing, radio, partnership with the education sector. This will require a better understanding of cultural beliefs, behaviour, knowledge, attitudes and practice.]	
50. Monitor the burden of **trauma-related vision loss** and advocate for context specific prevention strategies (e.g. reduce road traffic injuries and regulate the sale of fireworks).	
51. Engage positively with **traditional healers** around safe eye health practices. Alert people to the potential dangers of some traditional eye medicine.	
52. Integrate evidenced-based **primary eye care services for children** into general children's health services. Develop and implement sustainable **school eye health programmes**. Including screening and management for refractive error / amblyopia that are well-integrated within education services.	Refractive error
53. Develop models to encourage population demand and ensure access to accurate **refraction and affordable, good quality spectacles** (e.g. social entrepreneurship for presbyopic correction, public-private partnerships).	
54. Stop the **pandemic of high myopia**, through encouraging all children to spend time outside each day, developing technology to detect progressive myopia, identifying interventions to prevent it and control progression.	
55. Improve access to and quality of **rehabilitation services** for people with vision impairment. [Recruit / train eye care service providers, community-based volunteers, rehabilitation therapists, and psychosocial counsellors to add vision rehabilitation to the services they currently provide. Improve access to low vision assistive devices and good quality prosthetic eyes]	Rehabilitation
56. Ensure **children with low vision** can access good quality education and rehabilitation services that maximise their independence and educational opportunities.	
57. Strengthen understanding of the **impact of irreversible blindness and vision impairment on people's lives**. In order to ensure sufficient priority is given to their needs, to improve understanding of their capabilities, to increase awareness on the availability of accommodations and vision assistive equipment and to reduce stigma.	

58. Promote coordinated action to address key regional and global research priorities, to secure funding, make more rapid advances and reduce research waste through **global collaborative studies**.	Research
59. Develop **research capacity for eye health in low- and middle-income countries**, particularly for epidemiological, implementation and health systems research.	
60. Invest in high-quality **implementation science research** around how to effectively deliver, scale-up and sustain universal health coverage for eye health; building on interventions that are already known to work.<br>[Definition: Implementation science is the study of methods to promote the systematic uptake of clinical research findings and other evidence-based practices into sustained routine practice; it is interdisciplinary and needs to be multi-location for generalisability]	
61. Elucidate, through basic science research, the **underlying mechanisms of poorly understood eye diseases**, in order to develop new treatment approaches.	Research
62. Attract and support new **basic science research scientists** into the field of eyes and vision.	
63. Develop treatment approaches to **repair / regenerate the retina and central nervous system** for conditions where impaired vision is due to vascular or neurological damage (e.g. ischaemia, glaucoma).	
64. Research the human neural code for vision (study of information processing by nerve cells), to enable restoration of sight, through bionic and optogenetic technologies ("**artificial eyes**").	
65. Improve **procurement of equipment, medicines and consumables** to ensure availability, quality, and reduce cost.<br>(e.g. bulk purchasing, duties waiver, off-label medicines, regulations to ensure cost-effective options chosen)	Resources
66. Develop **local ophthalmic drug and device manufacturing** capacity to produce affordable medications e.g. via social enterprises.	
67. Develop local capacity to **procure and maintain sophisticated equipment**.	
68. Develop and implement evidence-based strategies for the effective **integration** of eye care at the **primary care level and with other medical services** (e.g child health, diabetes / NCD services); ensuring that services are widely accessible, affordable and of high quality, meeting the primary eye care needs of the population. This will require appropriate, robust, evidence-based clinical diagnosis and management strategies.	Systems
69. Develop evidence-based strategies for the effective **integration** of eye health services between the **primary** and **secondary / tertiary level** improving referral pathways; ensuring that there is recognition of those who need secondary level care and that there is a timely, reliable, accessible and affordable mechanism connecting people to the care they need.	
70. Reduce the **environmental impact** of providing eye care services, through improved practices. (e.g. decarbonize energy and supply chains, increase efficiency of practices worldwide, and develop a circular economy for eye care products such as equipment being re-used and recycled where appropriate).	
71. Develop **efficient, effective and productive eyecare systems** (with reduced waiting times) to respond to increased demand for services by a **growing and ageing population**.	
72. Strengthen **public-private partnerships** for delivery of services to contributing towards Universal Health Coverage for eye health.	
73. Develop regional and national mechanisms for **health technology assessment** (including system and patient pathway) to identify the most effective, efficient and appropriate diagnostic and therapeutic interventions.	Technology
74. Harness the potential for **big data and the application of artificial intelligence,** integrating them into eye health systems, with special attention to creating systems appropriate for low-resource settings.<br>To support diagnosis of conditions such as diabetic retinopathy, age related macular degeneration, glaucoma and retinopathy of prematurity. Establish ethical data governance frameworks, minimal dataset standards and compatible platforms that can be used nationally and internationally to create data bioresources that mean that every patient contact adds to our greater knowledge.	
75. **Develop affordable diagnostic technology** to take clinical measurements (visual function, intraocular pressure, anterior chamber depth), images (retina) and monitor conditions (e.g. via smart-phone, biosensors, wearables) in a primary care setting, improving access to services.	
76. Develop technology to support strengthened integration / referral systems, including **telemedicine and mHealth**.	
77. Innovate to **improve assistive technology** for people with low vision.	
78. Develop **sustained-release intraocular drugs** and **implant delivery systems** for a range of conditions (e.g AMD, uveitis).	Therapeutics
79. Develop a drug delivery approach that **penetrates the blood-ocular barrier** to help treat the most common intraocular diseases.	
80. Develop new **drugs to prevent fibrosis** in ocular surface and retinal scarring diseases.	
81. Realise the potential of the deepening understanding of the **genetic and molecular basis of eye disease** to develop diagnostics and precision/targeted interventions to preserve or restore vision.	
82. Develop a **vaccine for ocular chlamydia trachomatis** infection for long term control.	Trachoma
83. Develop and implement evidence-based enhanced **mass antibiotic distribution** strategies for trachoma control to more rapidly and reliably control chlamydia trachomatis infection.	
84. Develop and implement evidence-based strengthening of trachoma control strategies (**Facial cleanliness and Environmental improvement**) that more effectively limit the transmission of chlamydia trachomatis, and suppress the re-emergence of infection.	
85. Develop more effective assessment and management strategies for **uveitis**.	Uveitis

Regional List: Central Europe, Eastern Europe & Central Asia

This list is comprised of challenges ranked by participants from this region in the Top 10 in Round 2 ***or*** *Round 3.*
The top 5 challenges in Round 3 are bolded.

Identify and implement strategies to improve the quality, productivity, equity and access of cataract services.

Improve child eye health: integrate evidenced-based primary eye care services for children into general children's health services and ensure strong connections to secondary eye care services. Develop and implement sustainable school eye health programmes, including screening and management for refractive error / amblyopia, that are well-integrated within education services.

Develop and implement 'one-stop' services for people with diabetes, through integrating diabetic retinopathy screening services with general diabetes care and developing robust systems to ensure ongoing follow-up and referral for diabetic retinopathy assessment and treatment as appropriate.

Develop models to encourage population demand and ensure access to accurate refraction and affordable, good quality spectacles.

Strengthen the provision of retinopathy of prematurity (ROP) services at country level, for prevention, screening, treatment, follow-up and rehabilitation

Develop and implement effective, accessible and inexpensive pathway approaches for screening, diagnosing, monitoring and managing glaucoma.

Reduce sight loss from corneal infection, through promoting prevention and early detection strategies; strengthen the ability of ophthalmic and general health workers to identify and appropriately manage eye infections; make microbiological diagnosis and appropriate treatments more widely available.

Develop more effective, longer-lasting, safer and affordable treatments for diabetic retinopathy to halt progression and preserve vision (e.g. anti-VEGF therapies).

Develop and implement evidence-based strategies for the effective integration of eye care at the primary care level and with other medical services (e.g. child health, diabetes / NCD services); ensuring that services are widely accessible, affordable and of high quality, meeting the primary eye care needs of the population.

Develop competency-based training, responsive to local needs, including assessment and audit approaches to identify gaps and training needs of ophthalmic personnel and all relevant non-eye health care personnel to delineate roles and responsibilities of different cadres.

Develop updated short- and long-term global, regional and national strategies to follow Vision 2020

Develop affordable diagnostic technology to take clinical measurements (visual function, intraocular pressure, anterior chamber depth), images (retina) and monitor conditions (e.g. via smart-phone, biosensors, wearables) in a primary care setting, improving access to services.

Develop further effective prevention and long-lasting, low-cost treatment approaches for wet and dry AMD

Establish and implement minimum standards for eye health care for use around the world.

Identify and implement evidenced-based, effective, sustainable and context-relevant screening / early detection strategies for eye conditions.

Develop and implement evidence-based strategies for the effective integration of eye health services between the primary and secondary / tertiary level improving referral pathways; ensuring that there is recognition of those who need secondary level care and that there is a timely, reliable, accessible and affordable mechanism connecting people to the care they need.

Stop the pandemic of high myopia, through encouraging all children to spend time outside each day, developing technology to detect progressive myopia, identifying interventions to prevent it and control progression.

Regional List: High-Income Countries

This list is comprised of challenges ranked by participants from this region in the Top 10 in Round 2 ***or*** *Round 3.*
The top 5 challenges in Round 3 are bolded.

Develop models to encourage population demand and ensure access to accurate refraction and affordable, good quality spectacles.

Develop and implement services that prioritise, by design, reaching marginalised or vulnerable groups (women, poor communities, Indigenous people, ethnic minorities, people with disabilities, people in aged care, prisons, refugee camps) and people living in rural communities with quality, affordable eye services.

Identify and implement strategies to improve the quality, productivity, equity and access of cataract services.

Improve child eye health: integrate evidenced-based primary eye care services for children into general children's health services and ensure strong connections to secondary eye care services. Develop and implement sustainable school eye health programmes, including screening and management for refractive error / amblyopia, that are well-integrated within education services.

Increased support to geographical regions with particularly severe eye health human resource shortages, by international bodies, professional bodies/colleges, NGOs.

Develop and rigorously test strategies that reduce out of pocket costs for those requiring eye care who are unable to afford full-cost services e.g. subsidy, tiered pricing, insurance.

Develop and implement appropriately responsive programmes to increase the access to / use of eye health services and treatment. Through reducing barriers to accessing services and increasing demand through greater awareness of need and confidence in the health care provision.

Develop and implement 'one-stop' services for people with diabetes, through integrating diabetic retinopathy screening services with general diabetes care and developing robust systems to ensure ongoing follow-up and referral for diabetic retinopathy assessment and treatment as appropriate.

Develop and implement evidence-based strategies for the effective integration of eye health services between the primary and secondary / tertiary level improving referral pathways; ensuring that there is recognition of those who need secondary level care and that there is a timely, reliable, accessible and affordable mechanism connecting people to the care they need.

Identify and implement evidenced-based, effective, sustainable and context-relevant screening / early detection strategies for eye conditions.

Develop and implement evidence-based strategies for the effective integration of eye care at the primary care level and with other medical services (e.g. child health, diabetes / NCD services); ensuring that services are widely accessible, affordable and of high quality, meeting the primary eye care needs of the population.

Invest in high-quality implementation science research around how to effectively deliver, scale-up and sustain universal health coverage for eye health; building on interventions that are already known to work.

Encourage governments to prioritise delivering integrated patient-centred eye health care services for Universal Health Coverage.

Identify and implement evidenced-based, effective, sustainable and context-relevant health promotion / disease prevention strategies for eye health, that address underlying risk factors and lead to Healthy Ageing.

Harness the potential for big data and the application of artificial intelligence, integrating them into eye health systems, with special attention to creating systems appropriate for low-resource settings. To support diagnosis of conditions such as diabetic retinopathy, age related macular degeneration, glaucoma and retinopathy of prematurity. Establish ethical data governance frameworks, minimal dataset standards and compatible platforms that can be used nationally and internationally to create data bioresources that mean that every patient contact adds to our greater knowledge.

Develop affordable diagnostic technology to take clinical measurements (visual function, intraocular pressure, anterior chamber depth), images (retina) and monitor conditions (e.g. via smart-phone, biosensors, wearables) in a primary care setting, improving access to services.

Regional List: Latin America & Caribbean

*This list is comprised of challenges ranked by participants from this region in the Top 10 in Round 2 **or** Round 3.*
The top 5 challenges in Round 3 are bolded.

Improve child eye health: integrate evidenced-based primary eye care services for children into general children's health services and ensure strong connections to secondary eye care services. Develop and implement sustainable school eye health programmes, including screening and management for refractive error / amblyopia, that are well-integrated within education services.

Develop and implement evidence-based strategies for the effective integration of eye care at the primary care level and with other medical services (e.g. child health, diabetes / NCD services); ensuring that services are widely accessible, affordable and of high quality, meeting the primary eye care needs of the population.

Identify and implement strategies to improve the quality, productivity, equity and access of cataract services.

Identify and implement evidenced-based, effective, sustainable and context-relevant screening / early detection strategies for eye conditions.

Develop and implement 'one-stop' services for people with diabetes, through integrating diabetic retinopathy screening services with general diabetes care and developing robust systems to ensure ongoing follow-up and referral for diabetic retinopathy assessment and treatment as appropriate.

Develop models to encourage population demand and ensure access to accurate refraction and affordable, good quality spectacles.

Strengthen the provision of retinopathy of prematurity (ROP) services at country level, for prevention, screening, treatment, follow-up and rehabilitation

Develop efficient, effective and productive eyecare systems (with reduced waiting times) to respond to increased demand for services by a growing and ageing population.

Improve procurement of equipment, medicines and consumables to ensure availability, quality, and reduce cost.

Train more people in general, public health and sub-speciality ophthalmology and other cadres for the eye health workforce, particularly in regions where numbers of practitioners are limited. Strengthen and expand eye health training through online learning.

Ensure financing for eye health exists within national budgets / financing structures and increase the investment.

Strengthen leadership and public health expertise across all levels of eye health care, and ensure national level leadership has the ability to influence policy and resource allocation. To contribute to this process, strengthen regional and national professional bodies for eye health practitioners.

Establish and implement minimum standards for eye health care for use around the world.

Develop and implement services that prioritise, by design, reaching marginalised or vulnerable groups (women, poor communities, Indigenous people, ethnic minorities, people with disabilities, people in aged care, prisons, refugee camps) and people living in rural communities with quality, affordable eye services.

Encourage governments to prioritise delivering integrated patient-centred eye health care services for Universal Health Coverage.

Develop and implement evidence-based strategies for the effective integration of eye health services between the primary and secondary / tertiary level improving referral pathways; ensuring that there is recognition of those who need secondary level care and that there is a timely, reliable, accessible and affordable mechanism connecting people to the care they need.

Strengthen planning by national and regional governments for eye health human resource development.

Develop and implement appropriately responsive programmes to increase the access to / use of eye health services and treatment. Through reducing barriers to accessing services and increasing demand through greater awareness of need and confidence in the health care provision.

Regional List: North Africa & Middle East

This list is comprised of challenges ranked by participants from this region in the Top 10 in Round 2 ***or*** *Round 3.*
The top 5 challenges in Round 3 are bolded.

Develop and implement services that prioritise, by design, reaching marginalised or vulnerable groups (women, poor communities, Indigenous people, ethnic minorities, people with disabilities, people in aged care, prisons, refugee camps) and people living in rural communities with quality, affordable eye services.

Develop models to encourage population demand and ensure access to accurate refraction and affordable, good quality spectacles.

Identify and implement strategies to improve the quality, productivity, equity and access of cataract services.

Improve child eye health: integrate evidenced-based primary eye care services for children into general children's health services and ensure strong connections to secondary eye care services. Develop and implement sustainable school eye health programmes, including screening and management for refractive error / amblyopia, that are well-integrated within education services.

Train more people in general, public health and sub-speciality ophthalmology and other cadres for the eye health workforce, particularly in regions where numbers of practitioners are limited. Strengthen and expand eye health training through online learning.

Develop and implement 'one-stop' services for people with diabetes, through integrating diabetic retinopathy screening services with general diabetes care and developing robust systems to ensure ongoing follow-up and referral for diabetic retinopathy assessment and treatment as appropriate.

Develop and implement evidence-based strategies for the effective integration of eye health services between the primary and secondary / tertiary level improving referral pathways; ensuring that there is recognition of those who need secondary level care and that there is a timely, reliable, accessible and affordable mechanism connecting people to the care they need.

Develop more effective, longer-lasting, safer and affordable treatments for diabetic retinopathy to halt progression and preserve vision (e.g. anti-VEGF therapies).

Develop and implement evidence-based strategies for the effective integration of eye care at the primary care level and with other medical services (e.g. child health, diabetes / NCD services); ensuring that services are widely accessible, affordable and of high quality, meeting the primary eye care needs of the population.

Encourage governments to prioritise delivering integrated patient-centred eye health care services for Universal Health Coverage.

Develop and implement appropriately responsive programmes to increase the access to / use of eye health services and treatment. Through reducing barriers to accessing services and increasing demand through greater awareness of need and confidence in the health care provision.

Identify and implement evidenced-based, effective, sustainable and context-relevant screening / early detection strategies for eye conditions.

Strengthen the health information system (HIS) for eye health within health facilities, integrating them into national systems.

Strengthen leadership and public health expertise across all levels of eye health care, and ensure national level leadership has the ability to influence policy and resource allocation. To contribute to this process, strengthen regional and national professional bodies for eye health practitioners.

Regional List: South Asia

This list is comprised of challenges ranked by participants from this region in the Top 10 in Round 2 ***or*** *Round 3.*
The top 5 challenges in Round 3 are bolded.

Develop models to encourage population demand and ensure access to accurate refraction and affordable, good quality spectacles.

Identify and implement strategies to improve the quality, productivity, equity and access of cataract services.

Strengthen public-private partnerships for delivery of services to contributing towards Universal Health Coverage for eye health.

Develop and rigorously test strategies that reduce out of pocket costs for those requiring eye care who are unable to afford full-cost services e.g. subsidy, tiered pricing, insurance.

Develop and implement services that prioritise, by design, reaching marginalised or vulnerable groups (women, poor communities, Indigenous people, ethnic minorities, people with disabilities, people in aged care, prisons, refugee camps) and people living in rural communities with quality, affordable eye services.

Increased support to geographical regions with particularly severe eye health human resource shortages, by international bodies, professional bodies/colleges, NGOs.

Improve child eye health: integrate evidenced-based primary eye care services for children into general children's health services and ensure strong connections to secondary eye care services. Develop and implement sustainable school eye health programmes, including screening and management for refractive error / amblyopia, that are well-integrated within education services.

Identify and implement evidenced-based, effective, sustainable and context-relevant screening / early detection strategies for eye conditions.

Ensure financing for eye health exists within national budgets / financing structures and increase the investment.

Implement policies to recruit, retain and motivate ophthalmic personnel to deliver more productive services where they are most needed, including in the public sector or hard to reach communities e.g. through continuing professional development and transparent and participatory leadership and career progression plans at all levels.

Develop efficient, effective and productive eyecare systems (with reduced waiting times) to respond to increased demand for services by a growing and ageing population.

Encourage governments to prioritise delivering integrated patient-centred eye health care services for Universal Health Coverage.

Strengthen the health information system (HIS) for eye health within health facilities, integrating them into national systems.

Strengthen leadership and public health expertise across all levels of eye health care, and ensure national level leadership has the ability to influence policy and resource allocation. To contribute to this process, strengthen regional and national professional bodies for eye health practitioners.

Develop updated short- and long-term global, regional and national strategies to follow Vision 2020

Develop and implement evidence-based strategies for the effective integration of eye health services between the primary and secondary / tertiary level improving referral pathways; ensuring that there is recognition of those who need secondary level care and that there is a timely, reliable, accessible and affordable mechanism connecting people to the care they need.

Develop research capacity for eye health in low- and middle-income countries, particularly for epidemiological, implementation and health systems research.

Regional List: Southeast Asia, East Asia & Oceania

This list is comprised of challenges ranked by participants from this region in the Top 10 in Round 2 ***or*** *Round 3.*
The top 5 challenges in Round 3 are bolded.

Develop models to encourage population demand and ensure access to accurate refraction and affordable, good quality spectacles.

Improve child eye health: integrate evidenced-based primary eye care services for children into general children's health services and ensure strong connections to secondary eye care services. Develop and implement sustainable school eye health programmes, including screening and management for refractive error / amblyopia, that are well-integrated within education services.

Identify and implement strategies to improve the quality, productivity, equity and access of cataract services.

Develop and implement services that prioritise, by design, reaching marginalised or vulnerable groups (women, poor communities, Indigenous people, ethnic minorities, people with disabilities, people in aged care, prisons, refugee camps) and people living in rural communities with quality, affordable eye services.

Develop and rigorously test strategies that reduce out of pocket costs for those requiring eye care who are unable to afford full-cost services e.g. subsidy, tiered pricing, insurance.

Develop and implement 'one-stop' services for people with diabetes, through integrating diabetic retinopathy screening services with general diabetes care and developing robust systems to ensure ongoing follow-up and referral for diabetic retinopathy assessment and treatment as appropriate.

Develop and implement evidence-based strategies for the effective integration of eye health services between the primary and secondary / tertiary level improving referral pathways; ensuring that there is recognition of those who need secondary level care and that there is a timely, reliable, accessible and affordable mechanism connecting people to the care they need.

Develop technology to support strengthened integration / referral systems, including telemedicine and mHealth.

Increased support to geographical regions with particularly severe eye health human resource shortages, by international bodies, professional bodies/colleges, NGOs.

Develop and implement evidence-based strategies for the effective integration of eye care at the primary care level and with other medical services (e.g. child health, diabetes / NCD services); ensuring that services are widely accessible, affordable and of high quality, meeting the primary eye care needs of the population.

Strengthen leadership and public health expertise across all levels of eye health care, and ensure national level leadership has the ability to influence policy and resource allocation. To contribute to this process, strengthen regional and national professional bodies for eye health practitioners.

Stop the pandemic of high myopia, through encouraging all children to spend time outside each day, developing technology to detect progressive myopia, identifying interventions to prevent it and control progression.

Strengthen the health information system (HIS) for eye health within health facilities, integrating them into national systems.

Develop affordable diagnostic technology to take clinical measurements (visual function, intraocular pressure, anterior chamber depth), images (retina) and monitor conditions (e.g. via smart-phone, biosensors, wearables) in a primary care setting, improving access to services.

Develop and implement appropriately responsive programmes to increase the access to / use of eye health services and treatment. Through reducing barriers to accessing services and increasing demand through greater awareness of need and confidence in the health care provision.

Harness the potential for big data and the application of artificial intelligence, integrating them into eye health systems, with special attention to creating systems appropriate for low-resource settings. To support diagnosis of conditions such as diabetic retinopathy, age related macular degeneration, glaucoma and retinopathy of prematurity. Establish ethical data governance frameworks, minimal dataset standards and compatible platforms that can be used nationally and internationally to create data bioresources that mean that every patient contact adds to our greater knowledge.

Regional List: Sub-Saharan Africa

This list is comprised of challenges ranked by participants from this region in the Top 10 in Round 2 ***or*** *Round 3. The top 5 challenges in Round 3 are bolded.*

Identify and implement strategies to improve the quality, productivity, equity and access of cataract services.

Develop models to encourage population demand and ensure access to accurate refraction and affordable, good quality spectacles.

Develop and implement services that prioritise, by design, reaching marginalised or vulnerable groups (women, poor communities, Indigenous people, ethnic minorities, people with disabilities, people in aged care, prisons, refugee camps) and people living in rural communities with quality, affordable eye services.

Develop and implement appropriately responsive programmes to increase the access to / use of eye health services and treatment. Through reducing barriers to accessing services and increasing demand through greater awareness of need and confidence in the health care provision.

Increased support to geographical regions with particularly severe eye health human resource shortages, by international bodies, professional bodies/colleges, NGOs.

Develop and rigorously test strategies that reduce out of pocket costs for those requiring eye care who are unable to afford full-cost services e.g. subsidy, tiered pricing, insurance.

Develop and implement evidence-based strategies for the effective integration of eye health services between the primary and secondary / tertiary level improving referral pathways; ensuring that there is recognition of those who need secondary level care and that there is a timely, reliable, accessible and affordable mechanism connecting people to the care they need.

Improve child eye health: integrate evidenced-based primary eye care services for children into general children's health services and ensure strong connections to secondary eye care services. Develop and implement sustainable school eye health programmes, including screening and management for refractive error / amblyopia, that are well-integrated within education services.

Improve procurement of equipment, medicines and consumables to ensure availability, quality, and reduce cost.

Identify and implement evidenced-based, effective, sustainable and context-relevant screening / early detection strategies for eye conditions.

Implement policies to recruit, retain and motivate ophthalmic personnel to deliver more productive services where they are most needed, including in the public sector or hard to reach communities e.g. through continuing professional development and transparent and participatory leadership and career progression plans at all levels.

Develop and implement evidence-based strategies for the effective integration of eye care at the primary care level and with other medical services (e.g. child health, diabetes / NCD services); ensuring that services are widely accessible, affordable and of high quality, meeting the primary eye care needs of the population.

Ensure financing for eye health exists within national budgets / financing structures and increase the investment.

Develop and implement effective, accessible and inexpensive pathway approaches for screening, diagnosing, monitoring and managing glaucoma.

Build a stronger advocacy case for investing in eye health and communicate this more effectively. Through generating robust data on the importance of eye health in relation to sustainable development, quality of life, health in general, economics and the rapidly growing need for services. Work in a more coordinated and effective manner to communicate with governments, industry, philanthropists, donors, and the community about the importance of eye health.

Encourage governments to prioritise delivering integrated patient-centred eye health care services for Universal Health Coverage.

Strengthen leadership and public health expertise across all levels of eye health care, and ensure national level leadership has the ability to influence policy and resource allocation. To contribute to this process, strengthen regional and national professional bodies for eye health practitioners.

Develop research capacity for eye health in low- and middle-income countries, particularly for epidemiological, implementation and health systems research.

Challenge	Global							High-Income							South Asia							North Africa and Middle East						
	Round 3 Criteria					Rank		Round 3 Criteria					Rank		Round 3 Criteria					Rank		Round 3 Criteria					Rank	
	Disease burden reduction	Inequality reduction	Immediacy	Feasibility	Round 3 Combined Score	Round 3	Round 2	Disease burden reduction	Inequality reduction	Immediacy	Feasibility	Round 3 Combined Score	Round 3	Round 2	Disease burden reduction	Inequality reduction	Immediacy	Feasibility	Round 3 Combined Score	Round 3	Round 2	Disease burden reduction	Inequality reduction	Immediacy	Feasibility	Round 3 Combined Score	Round 3	Round 2
Encourage governments to prioritise delivering integrated people-centred eye health care services for Universal Health Coverage.	3.45	3.40	2.75	2.70	3.08	16	1	3.43	3.48	2.60	2.45	2.99	16	4	3.59	3.52	2.84	2.73	3.17	14	1	3.41	3.50	2.69	2.84	3.11	10	3
Improve child eye health: integrate evidence-based primary eye care services for children into general children's health services and ensure strong connections to secondary eye care services. Develop and implement sustainable school eye health programmes, including screening and management for refractive error / amblyopia, that are well-integrated within education services.	3.41	3.40	3.13	3.14	3.27	3	2	3.22	3.43	2.97	3.05	3.17	4	22	3.48	3.27	3.16	3.09	3.25	7	2	3.13	3.44	3.19	3.19	3.23	4	1
Ensure financing for eye health exists within national budgets / financing structures and increase the investı	3.54	3.38	3.03	2.53	3.12	13	3	3.50	3.38	2.91	2.36	3.04	12	25	3.68	3.41	3.09	2.66	3.21	9	7	3.50	3.31	2.97	2.59	3.09	12	22
Develop and implement evidence-based strategies for the effective integration of eye health services between the primary and secondary / tertiary level improving referral pathways; ensuring that there is recognition of those who need secondary level care and that there is a timely, reliable, accessible and affordable mechanism connecting people to the care they need.	3.48	3.32	3.01	2.93	3.18	7	4	3.43	3.26	2.86	2.83	3.09	9	9	2.91	3.48	3.25	3.07	3.18	13	8	3.34	3.31	3.09	2.84	3.15	7	2
Develop and implement services that prioritise, by design, reaching marginalised or vulnerable groups (women, poor communities, Indigenous people, ethnic minorities, people with disabilities, people in aged care, prisons, refugee camps) and people living in rural communities with quality, affordable eye services.	3.42	3.79	3.09	2.76	3.26	4	5	3.36	3.93	3.02	2.53	3.21	2	1	3.43	3.77	3.16	2.86	3.31	5	5	3.47	3.88	3.22	2.94	3.38	1	9
Strengthen leadership and public health expertise across all levels of eye health care, and ensure national level leadership has the ability to influence policy and resource allocation (including strengthening	3.24	3.06	2.68	2.89	2.97	28	6	3.02	2.88	2.50	2.78	2.79	37	26	3.36	3.00	2.89	3.09	3.09	23	4	3.22	2.97	2.56	2.84	2.90	29	10
Develop and implement evidence-based strategies for the effective integration of eye care at the primary care level and with other medical services (e.g. child health, diabetes / NCD services); ensuring that	3.47	3.44	2.94	2.85	3.18	8	7	3.34	3.31	2.84	2.69	3.05	10	5	3.43	3.48	2.84	2.91	3.16	15	23	3.31	3.41	3.03	2.72	3.12	9	11
Develop and implement appropriately responsive programmes to increase the access to / use of eye health services and treatment. e.g. reduce barriers to accessing services and increase demand through greater	3.42	3.41	2.93	2.88	3.16	11	8	3.34	3.53	2.84	2.83	3.14	7	2	3.52	3.32	2.93	2.86	3.16	16	15	3.25	3.47	2.91	2.63	3.06	15	5
Strengthen the health information system (HIS) for eye health within health facilities, integrating them into national systems.	3.10	2.95	2.66	2.80	2.88	34	9	2.97	2.79	2.60	2.74	2.78	39	14	3.25	3.02	2.73	2.70	2.93	33	3	3.00	2.97	2.66	2.78	2.85	34	7
Develop and implement effective, accessible and inexpensive pathway approaches for screening, diagnosing, monitoring and managing glaucoma.	3.47	3.13	2.96	2.61	3.04	21	10	3.50	3.03	2.93	2.50	2.99	16	17	3.48	2.91	2.75	2.64	2.94	31	33	3.22	3.03	3.03	2.53	2.95	23	12
Develop and implement evidence-based, effective, sustainable and context-relevant screening / early detection strategies for eye conditions.	3.51	3.27	2.95	2.93	3.17	10	11	3.48	3.26	2.62	2.83	3.05	10	7	3.68	3.30	3.07	2.84	3.22	8	12	3.44	3.16	2.84	2.78	3.05	16	6
Develop research capacity for eye health in low- and middle-income countries, particularly for epidemiological, implementation and health systems research.	3.12	3.05	2.49	2.85	2.88	33	12	3.09	3.17	2.34	2.69	2.82	34	27	3.18	2.93	2.55	2.80	2.86	36	9	2.97	2.88	2.53	2.72	2.77	37	34
Develop updated short- and long-term global, regional and national strategies to follow Vision 2020	3.18	3.04	2.60	3.14	2.99	26	13	2.95	3.02	2.45	3.07	2.87	29	32	3.43	3.14	2.73	3.16	3.11	20	6	2.91	2.81	2.47	2.84	2.76	38	19
Develop affordable diagnostic technology to take clinical measurements (visual function, intraocular pressure, anterior chamber depth), images (retina) and monitor conditions (e.g. via smart-phone, biosensors, wearables) in a primary care setting, improving access to services.	3.32	3.23	2.96	2.78	3.07	17	14	3.26	3.17	2.84	2.83	3.03	13	10	3.30	3.16	2.89	2.61	2.99	28	13	3.19	3.28	2.84	2.78	3.02	20	14
Develop models to encourage population demand and ensure access to accurate refraction and affordable, good quality spectacles.	3.54	3.37	3.35	3.14	3.35	1	15	3.33	3.34	3.24	3.05	3.24	1	11	3.66	3.34	3.45	3.39	3.46	1	26	3.47	3.44	3.41	3.00	3.33	2	16
Train more people in general, public health and sub-speciality ophthalmology and other cadres for the eye health workforce, particularly in regions where numbers of practitioners are limited. Strengthen and expand eye health training through online learning.	3.46	3.23	2.85	2.95	3.13	12	16	3.29	3.16	2.74	2.90	3.02	14	21	3.45	3.23	2.91	2.86	3.11	20	28	3.47	3.31	2.94	3.03	3.19	5	21
Harness the potential for big data and the application of artificial intelligence, integrating them into eye health systems, with special attention to creating systems appropriate for low-resource settings.	3.04	2.82	2.50	2.56	2.73	40	17	2.91	2.74	2.36	2.60	2.66	45	8	3.09	2.70	2.52	2.57	2.72	40	20	3.03	2.91	2.41	2.63	2.74	40	24
Stop the pandemic of high myopia, through encouraging all children to spend time outside each day, developing technology to detect progressive myopia, identifying interventions to prevent it and control	3.34	2.81	2.57	2.84	2.89	32	18	3.17	2.59	2.16	2.74	2.66	44	15	3.45	2.86	2.73	2.84	2.97	30	17	3.41	2.84	2.63	2.84	2.93	25	18
Strengthen planning by national and regional governments for eye health human resource development.	3.25	3.11	2.59	2.83	2.95	29	19	3.14	3.07	2.48	2.69	2.84	31	29	3.48	3.09	2.70	2.82	3.02	25	19	3.13	3.06	2.53	2.78	2.88	30	38
Invest in high-quality implementation science research around how to effectively deliver, scale-up and sustain universal health coverage for eye health; building on interventions that are already known to work.	3.28	3.07	2.53	2.81	2.92	31	20	3.41	3.14	2.57	2.91	3.01	15	3	3.41	3.11	2.68	2.73	2.98	29	11	2.88	2.69	2.25	2.75	2.64	45	39
Build a stronger advocacy case for investing in eye health and communicate this more effectively. Through generating robust data on the importance of eye health in relation to sustainable development, quality of life, health in general, economics and the rapidly growing need for services. Work in a more coordinated and effective manner to communicate with governments, industry, philanthropists, donors, and the community about the importance of eye health.	3.22	3.09	2.63	2.98	2.98	27	21	2.98	2.98	2.41	2.98	2.84	33	33	3.32	2.95	2.73	3.05	3.01	26	24	3.13	3.03	2.47	2.81	2.86	32	50
Establish and implement minimum standards for eye health care for use around the world.	3.12	3.06	2.66	2.89	2.93	30	22	3.02	3.21	2.55	2.78	2.89	26	23	3.20	3.02	2.89	2.93	3.01	26	14	3.06	2.88	2.53	2.97	2.86	32	31
Implement policies to recruit, retain and motivate ophthalmic personnel to deliver more productive services where they are most needed, including in the public sector or hard to reach communities.	3.38	3.33	2.94	2.75	3.10	15	23	3.17	3.16	2.90	2.72	2.99	18	42	3.59	3.43	3.02	2.77	3.20	10	29	3.16	3.25	2.84	2.78	3.01	22	17
Develop more effective, longer-lasting, safer and affordable treatments for diabetic retinopathy to halt progression and preserve vision (e.g. anti-VEGF therapies).	3.48	2.96	2.89	2.71	3.01	24	24	3.50	2.79	2.74	2.52	2.89	26	34	3.39	2.70	2.89	2.75	2.93	32	35	3.47	3.09	3.03	2.94	3.13	8	8
Develop and implement strategies that reduce out of pocket costs for those requiring eye care who are unable to afford full-cost services e.g. subsidy, tiered pricing, insurance.	3.41	3.66	3.12	2.65	3.21	5	25	3.31	3.74	3.12	2.47	3.16	6	40	3.57	3.75	3.14	2.86	3.33	4	32	3.22	3.59	3.00	2.59	3.10	11	23
Improve access to and quality of rehabilitation services for people with vision impairment, ensuring children with low vision can access support and good quality education, and adults are able to engage effectively in the workplace and society.	2.95	3.36	2.90	2.76	2.99	25	26	2.93	3.22	2.88	2.79	2.96	22	20	2.98	3.23	2.82	2.68	2.93	33	34	2.97	3.53	2.94	2.78	3.05	16	15
Identify and implement strategies to improve the quality, productivity, equity and access of cataract services.	3.63	3.43	3.23	3.07	3.34	2	27	3.43	3.19	3.05	3.03	3.18	3	30	3.82	3.50	3.25	3.18	3.44	2	37	3.44	3.47	3.38	3.00	3.32	3	27
Develop competency-based training, responsive to local needs, including assessment and audit approaches to identify gaps and training needs of ophthalmic personnel and all relevant non-eye health care personnel to delineate roles and responsibilities of different cadres.	3.32	3.02	2.81	3.03	3.05	20	28	3.09	2.98	2.69	2.97	2.93	25	43	3.48	3.09	3.02	2.98	3.14	17	30	3.16	2.94	2.63	2.91	2.91	27	42
Develop technology to support strengthened integration / referral systems, including telemedicine and mHealth.	3.22	3.12	2.99	2.91	3.06	19	29	3.05	2.97	2.86	3.00	2.97	21	13	3.43	3.27	3.07	2.95	3.18	12	27	2.97	3.00	2.94	2.88	2.95	23	28
Develop a more complete understanding of the epidemiology of eye disease across the life course (prevalence, distribution, incidence, risk factors) to support the development of approaches to prevent and slow progression of disease, and addressing poverty, literacy/education, living standards, access to clean air and water and sanitation.	3.18	2.94	2.30	2.83	2.81	37	30	3.00	3.02	2.05	2.81	2.72	43	28	3.32	2.91	2.36	2.80	2.85	37	21	3.19	2.75	2.25	2.59	2.70	43	13
Develop efficient, effective and productive eyecare systems (with reduced waiting times) to respond to increased demand for services by a growing and ageing population.	3.44	3.21	2.92	2.69	3.07	18	31	3.40	3.09	2.88	2.55	2.98	19	12	3.59	3.36	2.98	2.89	3.20	10	40	3.41	3.22	2.97	2.72	3.08	13	53
Improve procurement of equipment, medicines and consumables to ensure availability, quality, and reduce cost.	3.30	3.15	3.13	2.83	3.10	14	32	3.10	3.16	2.93	2.62	2.95	23	41	3.41	3.20	2.93	2.84	3.10	22	39	3.22	3.06	3.19	2.75	3.05	16	35
Develop an international standard core indicator set for eye health and eye care services, which can be used within a national statistics register and inter-country comparisons.	2.88	2.89	2.51	2.98	2.82	36	33	2.74	2.93	2.34	2.97	2.75	42	18	3.05	3.00	2.61	2.89	2.89	35	16	2.88	3.06	2.50	2.75	2.80	36	36
Develop high-quality economic analyses for eye health and improve the precision of estimates through global data collection collaborations. In order to make the case for increased resource allocation and to	3.01	2.92	2.51	2.89	2.83	35	34	2.88	2.90	2.28	2.98	2.76	40	31	3.07	2.70	2.59	2.77	2.78	39	18	2.94	2.75	2.47	2.75	2.73	41	46
Strengthen public-private partnerships for delivery of services to contributing towards Universal Health Coverage for eye health.	3.26	3.09	2.89	2.84	3.02	23	35	2.98	2.93	2.59	2.64	2.78	38	57	3.61	3.50	3.14	3.20	3.36	3	10	2.97	2.94	2.88	2.72	2.88	30	44
Develop and implement 'one-stop' services for people with diabetes, through integrating diabetic retinopathy screening services with general diabetes care and developing robust systems to ensure	3.49	3.16	3.10	2.95	3.18	8	36	3.43	3.09	2.95	2.95	3.10	8	44	3.39	3.09	3.11	2.89	3.12	18	31	3.44	3.19	3.19	2.94	3.19	5	41
Increased support to geographical regions with particularly severe eye health human resource shortages, by international bodies, professional bodies/colleges, NGOs.	3.41	3.44	3.06	2.85	3.19	6	37	3.38	3.53	3.03	2.71	3.16	5	47	3.66	3.45	3.16	2.91	3.30	6	38	3.06	3.41	2.94	2.88	3.07	14	26
Strengthen monitoring, audit and feedback on cataract and other surgical services to improve quality, including better use of technology and inclusion in national reporting.	3.20	2.96	2.92	3.01	3.02	22	38	2.81	2.76	2.79	3.02	2.84	31	66	3.36	2.93	2.91	3.07	3.07	24	50	3.25	3.13	2.91	2.84	3.03	19	43
Promote coordinated action to address key regional and global research priorities, to secure funding, make more rapid advances and reduce research waste through global collaborative studies.	3.01	2.85	2.51	2.84	2.80	38	39	3.09	2.98	2.36	2.81	2.81	35	19	2.93	2.84	2.68	2.93	2.85	37	36	2.84	2.63	2.38	2.81	2.66	44	54
Develop further effective prevention and long-lasting, low-cost treatment approaches for wet and dry AMD	3.18	2.75	2.62	2.39	2.73	39	40	3.52	2.76	2.76	2.36	2.85	30	16	3.00	2.68	2.48	2.48	2.66	41	47	3.22	2.75	2.66	2.38	2.75	39	33
Identify and implement evidenced-based, effective, sustainable and context-relevant health promotion / disease prevention strategies for eye health, that address underlying risk factors and lead to Healthy	.	.	.	.	.	.	42	3.00	2.84	2.34	2.84	2.76	40	6	.	.	.	.	.	.	.	3.03	3.00	2.50	2.88	2.85	34	32
Disaggregate all eye health data to monitor inequality between social groups (e.g. sex/gender, place of residence, socioeconomic status) and use the data to set targets to address inequities. - Disease burden	.	.	.	.	.	.	43	2.72	3.58	2.45	2.77	2.88	28	39	3.02	3.53	2.84	3.07	3.12	19	22	.	.	.	.	.	.	.
Implement a team-based service delivery model with clearly set roles and tasks across cadres, and improve the integration of optometrists, refractionists and other allied ophthalmic personnel.	.	.	.	.	.	.	46	3.17	2.97	2.86	2.91	2.98	19	36	.	.	.	.	.	.	.	3.19	2.84	2.88	2.75	2.91	27	30
Enable participation of people and communities with eye health problems in policy, practice and research development.	.	.	.	.	.	.	47	2.69	3.16	2.52	2.88	2.81	35	35	.	.	.	.	.	.	.	2.69	3.03	2.53	2.59	2.71	42	37
Strengthen the provision of retinopathy of prematurity (ROP) services at country level, for prevention, screening, treatment, follow-up and rehabilitation	.	.	.	.	.	.	49	.	.	.	.	.	.	.	.	.	.	.	.	.	.	3.19	2.97	3.13	2.81	3.02	20	40
Distribute spending in eye health in line with the relative magnitude of the problem, and the benefit / cost-benefit.	.	.	.	.	.	.	50	3.38	3.09	2.84	2.43	2.94	24	38	.	.	.	.	.	.	.	.	.	.	.	.	.	.
Reduce sight loss from corneal infection, through promoting prevention and early detection strategies; strengthen the ability of ophthalmic and general health workers to identify and appropriately manage eye	.	.	.	.	.	.	51	.	.	.	.	.	.	.	.	.	.	.	.	.	.	.	.	.	.	.	.	.
Create effective and sustainable networks of eye banks within each country, including donor supply and corneal transplant services.	.	.	.	.	.	.	53	.	.	.	.	.	.	.	.	.	.	.	.	.	.	.	.	.	.	.	.	.
Increase understanding of the association between vision impairment and quality of life, aging, dementia, depression, functional decline and mortality and the impact of treatment and vision rehabilitation on	.	.	.	.	.	.	54	2.66	2.74	2.21	2.86	2.62	46	37	.	.	.	.	.	.	.	.	.	.	.	.	.	.
Develop population-specific risk calculators to provide risk predictions for the development and progression of specific eye conditions and guide the provision of screening, monitoring and treatment	.	.	.	.	.	.	59	.	.	.	.	.	.	.	.	.	.	.	.	.	.	.	.	.	.	.	.	.
Reduce the environmental impact of providing eye care services, through improved practices. (e.g. decarbonize energy and supply chains, increase efficiency of practices worldwide, and develop a circular	.	.	.	.	.	.	61	.	.	.	.	.	.	.	.	.	.	.	.	.	.	.	.	.	.	.	.	.
Develop regional and national mechanisms for health technology assessment (including system and patient pathway) to identify the most effective, efficient and appropriate diagnostic and therapeutic	.	.	.	.	.	.	64	.	.	.	.	.	.	.	.	.	.	.	.	.	.	.	.	.	.	.	.	.
Develop treatment approaches to repair / regenerate the retina and central nervous system for conditions where impaired vision is due to vascular or neurological damage (e.g. ischaemia, glaucoma).	.	.	.	.	.	.	67	.	.	.	.	.	.	.	.	.	.	.	.	.	.	.	.	.	.	.	.	.
Develop new treatments to rehabilitate vision from corneal blindness for example developing tissue-engineered corneas for transplantation (artificial corneas) and enable corneal limbal regeneration.	.	.	.	.	.	.	68	.	.	.	.	.	.	.	.	.	.	.	.	.	.	.	.	.	.	.	.	.

Challenge	Sub-Saharan Africa							Latin America & Caribbean							Southeast Asia, East Asia & Oceania							Central Europe, Eastern Europe & Central Asia						
	Round 3 Criteria					Rank		Round 3 Criteria					Rank		Round 3 Criteria					Rank		Round 3 Criteria					Rank	
	Disease burden reduction	Inequality reduction	Immediacy	Feasibility	Round 3 Combined Score	Round 3	Round 2	Disease burden reduction	Inequality reduction	Immediacy	Feasibility	Round 3 Combined Score	Round 3	Round 2	Disease burden reduction	Inequality reduction	Immediacy	Feasibility	Round 3 Combined Score	Round 3	Round 2	Disease burden reduction	Inequality reduction	Immediacy	Feasibility	Round 3 Combined Score	Round 3	Round 2
Encourage governments to prioritise delivering integrated people-centred eye health care services for Universal Health Coverage.	3.43	3.37	2.80	2.83	3.11	22	2	3.54	3.34	2.97	2.74	3.15	22	6	3.45	3.40	2.67	2.69	3.05	14	13	3.21	2.95	2.68	2.47	2.83	29	50
Improve child eye health: integrate evidence-based primary eye care services for children into general children's health services and ensure strong connections to secondary eye care services. Develop and implement sustainable school eye health programmes, including screening and management for refractive error / amblyopia, that are well-integrated within education services.	3.48	3.38	3.10	3.15	3.28	8	6	3.63	3.51	3.29	3.14	3.39	1	2	3.48	3.40	3.14	3.21	3.31	2	10	3.42	3.47	3.26	3.21	3.34	2	1
Ensure financing for eye health exists within national budgets / financing structures and increase the investi	3.62	3.47	3.13	2.56	3.19	14	1	3.60	3.46	3.03	2.66	3.19	14	1	3.50	3.26	3.02	2.55	3.08	13	29	3.00	3.00	2.79	2.26	2.76	34	20
Develop and implement evidence-based strategies for the effective integration of eye health services between the primary and secondary / tertiary level improving referral pathways; ensuring that there is recognition of those who need secondary level care and that there is a timely, reliable, accessible and affordable mechanism connecting people to the care they need.	3.61	3.43	3.12	3.03	3.30	7	3	3.54	3.29	3.06	2.83	3.18	16	7	3.50	3.36	2.90	2.98	3.18	7	11	3.05	3.00	2.84	2.79	2.92	22	9
Develop and implement services that prioritise, by design, reaching marginalised or vulnerable groups (women, poor communities, Indigenous people, ethnic minorities, people with disabilities, people in aged care, prisons, refugee camps) and people living in rural communities with quality, affordable eye services.	3.54	3.81	3.13	2.86	3.33	3	14	3.40	3.60	3.09	2.66	3.19	14	5	3.29	3.88	3.00	2.71	3.22	4	3	3.25	3.35	2.85	2.60	3.01	12	42
Strengthen leadership and public health expertise across all levels of eye health care, and ensure national level leadership has the ability to influence policy and resource allocation (including strengthening	3.33	3.11	2.73	2.89	3.01	28	8	3.40	3.34	2.89	2.89	3.13	26	3	3.12	3.19	2.55	3.00	2.96	20	1	3.11	2.79	2.63	2.58	2.78	32	39
Develop and implement evidence-based strategies for the effective integration of eye care at the primary care level and with other medical services (e.g. child health, diabetes / NCD services); ensuring that	3.61	3.50	2.94	2.90	3.24	12	4	3.60	3.63	3.29	3.00	3.38	2	13	3.43	3.45	2.88	2.76	3.13	10	6	3.37	3.05	2.84	3.00	3.07	9	23
Develop and implement appropriately responsive programmes to increase the access to / use of eye health services and treatment. e.g. reduce barriers to accessing services and increase demand through greater	3.56	3.49	3.10	3.11	3.31	4	16	3.43	3.49	3.06	2.86	3.21	12	10	3.36	3.26	2.69	2.74	3.01	15	7	3.10	2.85	2.70	2.60	2.81	31	17
Strengthen the health information system (HIS) for eye health within health facilities, integrating them into national systems.	3.16	2.99	2.65	2.94	2.94	32	12	3.23	3.09	2.74	2.74	2.95	39	12	3.05	3.02	2.69	2.88	2.91	27	4	2.75	2.50	2.50	2.38	2.53	43	49
Develop and implement effective, accessible and inexpensive pathway approaches for screening, diagnosing, monitoring and managing glaucoma.	3.61	3.29	3.01	2.73	3.16	16	5	3.57	3.31	3.17	2.63	3.17	17	11	3.17	2.95	2.69	2.52	2.83	34	31	3.48	3.29	3.24	2.57	3.14	6	2
Develop and implement evidence-based, effective, sustainable and context-relevant screening / early detection strategies for eye conditions.	3.53	3.40	3.02	3.03	3.25	10	22	3.57	3.40	3.26	3.06	3.32	4	31	3.43	3.07	3.00	2.98	3.12	11	12	3.29	2.95	2.86	2.90	3.00	13	8
Develop research capacity for eye health in low- and middle-income countries, particularly for epidemiological, implementation and health systems research.	3.14	3.08	2.53	3.05	2.95	31	10	3.29	3.06	2.54	2.80	2.92	41	19	3.02	3.10	2.26	2.90	2.82	36	25	3.11	3.00	2.95	2.63	2.92	22	19
Develop updated short- and long-term global, regional and national strategies to follow Vision 2020	3.29	3.11	2.68	3.27	3.09	23	13	3.46	3.26	2.86	3.06	3.16	21	32	2.95	2.83	2.36	3.21	2.84	32	19	3.16	3.00	2.68	3.11	2.99	15	3
Develop affordable diagnostic technology to take clinical measurements (visual function, intraocular pressure, anterior chamber depth), images (retina) and monitor conditions (e.g. via smart-phone, biosensors, wearables) in a primary care setting, improving access to services.	3.34	3.26	3.05	2.80	3.11	20	31	3.46	3.31	3.06	2.83	3.16	19	34	3.43	3.24	2.98	2.76	3.10	12	5	3.24	3.10	3.05	2.81	3.05	11	4
Develop models to encourage population demand and ensure access to accurate refraction and affordable, good quality spectacles.	3.65	3.37	3.39	3.17	3.40	2	23	3.51	3.46	3.29	2.94	3.30	6	14	3.60	3.38	3.31	3.31	3.40	1	8	3.26	3.21	3.32	2.95	3.18	3	31
Train more people in general, public health and sub-speciality ophthalmology and other cadres for the eye health workforce, particularly in regions where numbers of practitioners are limited. Strengthen and expand eye health training through online learning.	3.58	3.30	2.92	3.08	3.22	13	11	3.57	3.43	3.06	2.91	3.24	10	20	3.43	3.12	2.52	2.83	2.98	17	32	3.21	2.89	2.89	2.84	2.96	17	35
Harness the potential for big data and the application of artificial intelligence, integrating them into eye health systems, with special attention to creating systems appropriate for low-resource settings.	3.08	2.84	2.45	2.48	2.71	40	30	3.20	3.11	2.69	2.31	2.83	44	35	3.05	2.81	2.62	2.79	2.82	37	9	2.81	2.63	2.75	2.63	2.70	37	28
Stop the pandemic of high myopia, through encouraging all children to spend time outside each day, developing technology to detect progressive myopia, identifying interventions to prevent it and control	3.33	2.88	2.65	2.88	2.93	33	38	3.37	2.97	2.69	2.83	2.96	36	23	3.43	2.69	2.50	2.81	2.86	30	2	3.24	2.86	2.86	3.00	2.99	14	10
Strengthen planning by national and regional governments for eye health human resource development.	3.38	3.21	2.71	3.04	3.08	25	20	3.23	3.20	2.63	2.77	2.96	38	8	3.02	3.00	2.31	2.76	2.77	38	14	3.11	2.95	2.68	2.53	2.82	30	18
Invest in high-quality implementation science research around how to effectively deliver, scale-up and sustain universal health coverage for eye health; building on interventions that are already known to work.	3.33	3.11	2.60	2.89	2.98	30	32	3.46	3.17	2.63	2.60	2.96	36	37	3.14	3.12	2.31	2.88	2.86	29	23	2.95	2.95	2.53	2.58	2.75	35	29
Build a stronger advocacy case for investing in eye health and communicate this more effectively. Through generating robust data on the importance of eye health in relation to sustainable development, quality of life, health in general, economics and the rapidly growing need for services. Work in a more coordinated and effective manner to communicate with governments, industry, philanthropists, donors, and the community about the importance of eye health.	3.37	3.21	2.75	3.11	3.11	21	7	3.40	3.37	2.91	2.89	3.14	23	46	3.10	2.93	2.43	2.93	2.85	31	15	2.95	2.95	2.58	2.63	2.78	32	45
Establish and implement minimum standards for eye health care for use around the world.	3.13	3.01	2.62	2.89	2.91	35	37	3.31	3.11	2.83	3.00	3.06	28	4	3.07	3.17	2.64	2.90	2.95	22	18	3.05	2.89	2.63	2.79	2.84	28	7
Implement policies to recruit, retain and motivate ophthalmic personnel to deliver more productive services where they are most needed, including in the public sector or hard to reach communities.	3.60	3.51	3.01	2.83	3.24	11	9	3.37	3.29	3.00	2.89	3.14	25	29	3.24	3.24	2.79	2.55	2.95	21	38	3.11	3.00	2.84	2.58	2.88	25	40
Develop more effective, longer-lasting, safer and affordable treatments for diabetic retinopathy to halt progression and preserve vision (e.g. anti-VEGF therapies).	3.50	3.04	2.85	2.71	3.02	27	18	3.66	3.31	3.09	2.71	3.19	13	22	3.38	2.79	2.83	2.71	2.93	24	30	3.38	3.05	3.05	2.86	3.08	8	6
Develop and implement strategies that reduce out of pocket costs for those requiring eye care who are unable to afford full-cost services e.g. subsidy, tiered pricing, insurance.	3.54	3.71	3.20	2.74	3.30	6	28	3.51	3.54	3.09	2.54	3.17	17	17	3.38	3.69	3.17	2.64	3.22	4	16	2.90	3.25	2.85	2.55	2.89	24	52
Improve access to and quality of rehabilitation services for people with vision impairment, ensuring children with low vision can access support and good quality education, and adults are able to engage effectively in the workplace and society.	2.87	3.40	2.91	2.83	3.00	29	25	3.29	3.57	3.17	2.63	3.16	19	24	2.79	3.40	2.76	2.74	2.92	25	55	3.05	3.05	2.90	2.80	2.95	18	48
Identify and implement strategies to improve the quality, productivity, equity and access of cataract services.	3.73	3.49	3.32	3.15	3.42	1	17	3.74	3.60	3.23	2.91	3.37	3	39	3.60	3.31	3.10	2.90	3.23	3	39	3.53	3.53	3.32	3.26	3.41	1	15
Develop competency-based training, responsive to local needs, including assessment and audit approaches to identify gaps and training needs of ophthalmic personnel and all relevant non-eye health care personnel to delineate roles and responsibilities of different cadres.	3.44	3.03	2.93	3.18	3.15	18	26	3.37	3.09	2.80	2.86	3.03	33	16	3.29	3.02	2.57	3.02	2.98	17	28	3.26	3.00	2.95	3.05	3.07	9	11
Develop technology to support strengthened integration / referral systems, including telemedicine and mHealth.	3.27	3.13	3.04	2.86	3.07	26	39	3.29	3.20	3.03	2.91	3.11	27	21	3.36	3.21	3.02	2.98	3.14	8	37	3.05	3.05	2.86	2.81	2.94	19	14
Develop a more complete understanding of the epidemiology of eye disease across the life course (prevalence, distribution, incidence, risk factors) to support the development of approaches to prevent and slow progression of disease, and addressing poverty, literacy/education, living standards, access to clean air and water and sanitation.	3.30	3.04	2.39	2.84	2.89	36	34	3.46	3.14	2.69	2.80	3.02	34	27	2.88	2.79	2.07	3.14	2.72	42	57	2.84	2.47	2.32	2.68	2.58	40	16
Develop efficient, effective and productive eyecare systems (with reduced waiting times) to respond to increased demand for services by a growing and ageing population.	3.47	3.25	2.97	2.70	3.10	24	41	3.60	3.49	3.23	2.77	3.27	8	9	3.29	2.95	2.55	2.57	2.84	32	17	3.20	3.00	2.85	2.70	2.94	20	47
Improve procurement of equipment, medicines and consumables to ensure availability, quality, and reduce cost.	3.51	3.19	3.35	2.99	3.26	9	15	3.46	3.40	3.23	2.91	3.25	9	47	3.07	3.00	3.07	2.74	2.97	19	41	2.95	2.75	2.90	2.80	2.85	27	30
Develop an international standard core indicator set for eye health and eye care services, which can be used within a national statistics register and inter-country comparisons.	2.95	2.83	2.58	3.14	2.88	39	48	3.09	3.06	2.63	2.94	2.93	40	28	2.62	2.74	2.36	2.93	2.66	44	22	2.75	2.44	2.63	3.00	2.70	37	26
Develop high-quality economic analyses for eye health and improve the precision of estimates through global data collection collaborations. In order to make the case for increased resource allocation and to	3.09	2.98	2.63	2.97	2.92	34	36	3.17	3.26	2.77	2.83	3.01	35	43	2.88	2.95	2.26	2.90	2.75	39	20	2.89	2.74	2.53	2.79	2.74	36	61
Strengthen public-private partnerships for delivery of services to contributing towards Universal Health Coverage for eye health.	3.39	3.15	3.04	2.91	3.13	19	24	3.51	3.37	3.09	2.89	3.21	11	44	3.14	2.79	2.74	2.86	2.88	28	47	2.84	2.74	2.47	2.32	2.59	39	62
Develop and implement 'one-stop' services for people with diabetes, through integrating diabetic retinopathy screening services with general diabetes care and developing robust systems to ensure	3.55	3.14	3.03	2.98	3.18	15	33	3.66	3.43	3.34	2.80	3.31	5	33	3.50	3.17	3.10	3.07	3.21	6	42	3.42	3.11	3.32	2.89	3.18	3	38
Increased support to geographical regions with particularly severe eye health human resource shortages, by international bodies, professional bodies/colleges, NGOs.	3.57	3.51	3.13	2.95	3.29	5	29	3.26	3.31	3.11	2.89	3.14	23	53	3.38	3.43	2.93	2.81	3.14	9	34	3.00	3.05	2.89	2.58	2.88	25	43
Strengthen monitoring, audit and feedback on cataract and other surgical services to improve quality, including better use of technology and inclusion in national reporting.	3.37	3.05	3.05	3.15	3.15	16	19	3.31	3.06	2.91	2.86	3.04	32	41	3.07	2.90	2.81	2.88	2.92	26	26	3.00	2.88	2.88	2.94	2.92	21	21
Promote coordinated action to address key regional and global research priorities, to secure funding, make more rapid advances and reduce research waste through global collaborative studies.	3.10	2.88	2.61	2.89	2.87	38	53	3.17	3.03	2.69	2.77	2.91	42	42	2.93	2.76	2.33	2.88	2.73	40	27	2.74	2.47	2.32	2.53	2.51	44	32
Develop further effective prevention and long-lasting, low-cost treatment approaches for wet and dry AMD	3.01	2.67	2.44	2.36	2.62	41	73	3.26	2.91	2.77	2.37	2.83	44	54	3.17	2.69	2.69	2.36	2.73	40	51	3.24	3.14	2.95	2.57	2.98	16	5
Identify and implement evidenced-based, effective, sustainable and context-relevant health promotion / disease prevention strategies for eye health, that address underlying risk factors and lead to Healthy	.	.	.	.	.	.	.	3.40	3.17	2.74	2.86	3.04	31	40	3.10	2.93	2.31	3.00	2.83	34	40	.	.	.	.	.	.	.
Disaggregate all eye health data to monitor inequality between social groups (e.g. sex/gender, place of residence, socioeconomic status) and use the data to set targets to address inequities. - Disease burden	.	.	.	.	.	.	.	3.03	3.59	2.71	2.88	3.05	30	25	2.67	3.60	2.57	2.90	2.93	23	21	.	.	.	.	.	.	.
Implement a team-based service delivery model with clearly set roles and tasks across cadres, and improve the integration of optometrists, refractionists and other allied ophthalmic personnel.	.	.	.	.	.	.	.	3.43	3.17	2.91	2.74	3.06	28	26	3.26	2.93	2.79	3.07	3.01	15	35	.	.	.	.	.	.	.
Enable participation of people and communities with eye health problems in policy, practice and research development.	2.98	3.17	2.64	2.78	2.89	37	40	3.06	3.11	2.57	2.74	2.87	43	30	.	.	.	.	.	.	.	.	.	.	.	.	.	.
Strengthen the provision of retinopathy of prematurity (ROP) services at country level, for prevention, screening, treatment, follow-up and rehabilitation	.	.	.	.	.	.	.	3.54	3.29	3.37	2.94	3.29	7	15	.	.	.	.	.	.	.	3.33	3.14	3.19	2.95	3.155	5	22
Distribute spending in eye health in line with the relative magnitude of the problem, and the benefit / cost-benefit.	.	.	.	.	.	.	.	.	.	.	.	.	.	.	.	.	.	.	.	.	.	.	.	.	.	.	.	.
Reduce sight loss from corneal infection, through promoting prevention and early detection strategies; strengthen the ability of ophthalmic and general health workers to identify and appropriately manage eye	.	.	.	.	.	.	.	.	.	.	.	.	.	.	.	.	.	.	.	.	.	3.32	3.05	3.05	3.00	3.11	7	34
Create effective and sustainable networks of eye banks within each country, including donor supply and corneal transplant services.	.	.	.	.	.	.	.	.	.	.	.	.	.	.	.	.	.	.	.	.	.	2.79	2.58	2.68	2.26	2.58	40	12
Increase understanding of the association between vision impairment and quality of life, aging, dementia, depression, functional decline and mortality and the impact of treatment and vision rehabilitation on	.	.	.	.	.	.	.	.	.	.	.	.	.	.	.	.	.	.	.	.	.	.	.	.	.	.	.	.
Develop population-specific risk calculators to provide risk predictions for the development and progression of specific eye conditions and guide the provision of screening, monitoring and treatment	.	.	.	.	.	.	.	.	.	.	.	.	.	.	.	.	.	.	.	.	.	2.63	2.37	2.32	2.58	2.47	45	36
Reduce the environmental impact of providing eye care services, through improved practices. (e.g. decarbonize energy and supply chains, increase efficiency of practices worldwide, and develop a circular	.	.	.	.	.	.	.	.	.	.	.	.	.	.	2.17	2.26	1.98	2.45	2.21	45	33	.	.	.	.	.	.	.
Develop regional and national mechanisms for health technology assessment (including system and patient pathway) to identify the most effective, efficient and appropriate diagnostic and therapeutic	.	.	.	.	.	.	.	.	.	.	.	.	.	.	2.88	2.71	2.50	2.64	2.68	43	36	.	.	.	.	.	.	.
Develop treatment approaches to repair / regenerate the retina and central nervous system for conditions where impaired vision is due to vascular or neurological damage (e.g. ischaemia, glaucoma).	.	.	.	.	.	.	.	.	.	.	.	.	.	.	.	.	.	.	.	.	.	3.11	2.63	2.42	2.16	2.58	40	37
Develop new treatments to rehabilitate vision from corneal blindness for example developing tissue-engineered corneas for transplantation (artificial corneas) and enable corneal limbal regeneration.	.	.	.	.	.	.	.	.	.	.	.	.	.	.	.	.	.	.	.	.	.	2.84	2.53	2.21	2.11	2.42	46	33

Top 10 priorities for the two regions with the highest proportion of participants non-residents from high income countries

Rank: All 42 participants	Rank: 21 permanent residents	Top 10 priorities after Round 3 Southeast Asia, East Asia and Oceania
1	1	Develop models to encourage population demand and ensure access to accurate refraction and affordable, good quality spectacles.
2	2	Improve child eye health: integrate evidenced-based primary eye care services for children into general children's health services and ensure strong connections to secondary eye care services. Develop and implement sustainable school eye health programmes, including screening and management for refractive error / amblyopia, that are well-integrated within education services.
3	8	Identify and implement strategies to improve the quality, productivity, equity and access of cataract services.
4	6	Develop and implement services that prioritise, by design, reaching marginalised or vulnerable groups (women, poor communities, Indigenous people, ethnic minorities, people with disabilities, people in aged care, prisons, refugee camps) and people living in rural communities with quality, affordable eye services.
5	11	Develop and rigorously test strategies that reduce out of pocket costs for those requiring eye care who are unable to afford full-cost services e.g. subsidy, tiered pricing, insurance.
6	12	Develop and implement 'one-stop' services for people with diabetes, through integrating diabetic retinopathy screening services with general diabetes care and developing robust systems to ensure ongoing follow-up and referral for diabetic retinopathy assessment and treatment as appropriate.
7	9	Develop and implement evidence-based strategies for the effective integration of eye health services between the primary and secondary / tertiary level improving referral pathways; ensuring that there is recognition of those who need secondary level care and that there is a timely, reliable, accessible and affordable mechanism connecting people to the care they need.
8	7	Develop technology to support strengthened integration / referral systems, including telemedicine and mHealth.
9	3	Increased support to geographical regions with particularly severe eye health human resource shortages, by international bodies, professional bodies/colleges, NGOs.
10	4	Develop and implement evidence-based strategies for the effective integration of eye care at the primary care level and with other medical services (e.g. child health, diabetes / NCD services); ensuring that services are widely accessible, affordable and of high quality, meeting the primary eye care needs of the population.

Rank: All 104 participants	Rank: 74 permanent residents	Top 10 priorities after Round 3 Sub-Saharan Africa
1	6	Identify and implement strategies to improve the quality, productivity, equity and access of cataract services.
2	2	Develop models to encourage population demand and ensure access to accurate refraction and affordable, good quality spectacles.
3	1	Develop and implement services that prioritise, by design, reaching marginalised or vulnerable groups (women, poor communities, Indigenous people, ethnic minorities, people with disabilities, people in aged care, prisons, refugee camps) and people living in rural communities with quality, affordable eye services.
4	3	Develop and implement appropriately responsive programmes to increase the access to / use of eye health services and treatment. Through reducing barriers to accessing services and increasing demand through greater awareness of need and confidence in the health care provision.
5	4	Increased support to geographical regions with particularly severe eye health human resource shortages, by international bodies, professional bodies/colleges, NGOs.
6	10	Develop and rigorously test strategies that reduce out of pocket costs for those requiring eye care who are unable to afford full-cost services e.g. subsidy, tiered pricing, insurance.
7	5	Develop and implement evidence-based strategies for the effective integration of eye health services between the primary and secondary / tertiary level improving referral pathways; ensuring that there is recognition of those who need secondary level care and that there is a timely, reliable, accessible and affordable mechanism connecting people to the care they need.
8	8	Improve child eye health: integrate evidenced-based primary eye care services for children into general children's health services and ensure strong connections to secondary eye care services. Develop and implement sustainable school eye health programmes, including screening and management for refractive error / amblyopia, that are well-integrated within education services.
9	7	Improve procurement of equipment, medicines and consumables to ensure availability, quality, and reduce cost.
10	12	Identify and implement evidenced-based, effective, sustainable and context-relevant screening / early detection strategies for eye conditions.
